# Application of herbs and active ingredients ameliorate non-alcoholic fatty liver disease under the guidance of traditional Chinese medicine

**DOI:** 10.3389/fendo.2022.1000727

**Published:** 2022-09-20

**Authors:** Zhijia Zhou, Jinghao Zhang, Liping You, Tao Wang, Kaixia Wang, Lingtai Wang, Xiaoni Kong, Yueqiu Gao, Xuehua Sun

**Affiliations:** ^1^ Department of Hepatology, ShuGuang Hospital Affiliated to Shanghai University of Traditional Chinese Medicine, Shanghai, China; ^2^ Department of Infection, Oriental Hospital Affiliated to Tongji University, Shanghai, China; ^3^ Central Laboratory, ShuGuang Hospital Affiliated to Shanghai University of Traditional Chinese Medicine, Shanghai, China

**Keywords:** Dampness-heat syndrome, TCM, non-alcoholic fatty liver disease (NAFLD), herbs, ingredients

## Abstract

Non-alcoholic fatty liver disease (NAFLD) is a global health problem, and its prevalence has been on the rise in recent years. Traditional Chinese Medicine (TCM) contains a wealth of therapeutic resources and has been in use for thousands of years regarding the prevention of liver disease and has been shown to be effective in the treatment of NAFLD in China. but the molecular mechanisms behind it have not been elucidated. In this article, we have updated and summarized the research and evidence concerning herbs and their active ingredients for the treatment *in vivo* and *vitro* models of NAFLD or NASH, by searching PubMed, Web of Science and SciFinder databases. In particular, we have found that most of the herbs and active ingredients reported so far have the effect of clearing heat and dispelling dampness, which is consistent with the concept of dampness-heat syndrome, in TCM theory. we have attempted to establish the TCM theory and modern pharmacological mechanisms links between herbs and monomers according to their TCM efficacy, experiment models, targets of modulation and amelioration of NAFLD pathology. Thus, we provide ideas and perspectives for further exploration of the pathogenesis of NAFLD and herbal therapy, helping to further the scientific connotation of TCM theories and promote the modernization of TCM.

## 1 Introduction

Non-alcoholic fatty liver disease (NAFLD) is defined as hepatic steatosis, excluding the causes of significant alcohol consumption, steatogenic medication or hereditary disorders (1). It is histologically characterized by steatosis (2). About 25% of adults worldwide suffer from NAFLD (3) and despite the known risk factors such as diabetes, obesity, age, gender and race, the prevalence of NAFLD is still increasing (4). NAFLD elevates the risk of all-cause mortality, liver-related deaths, malignancy, diabetes and coronary artery disease (5).

**Figure 1:**
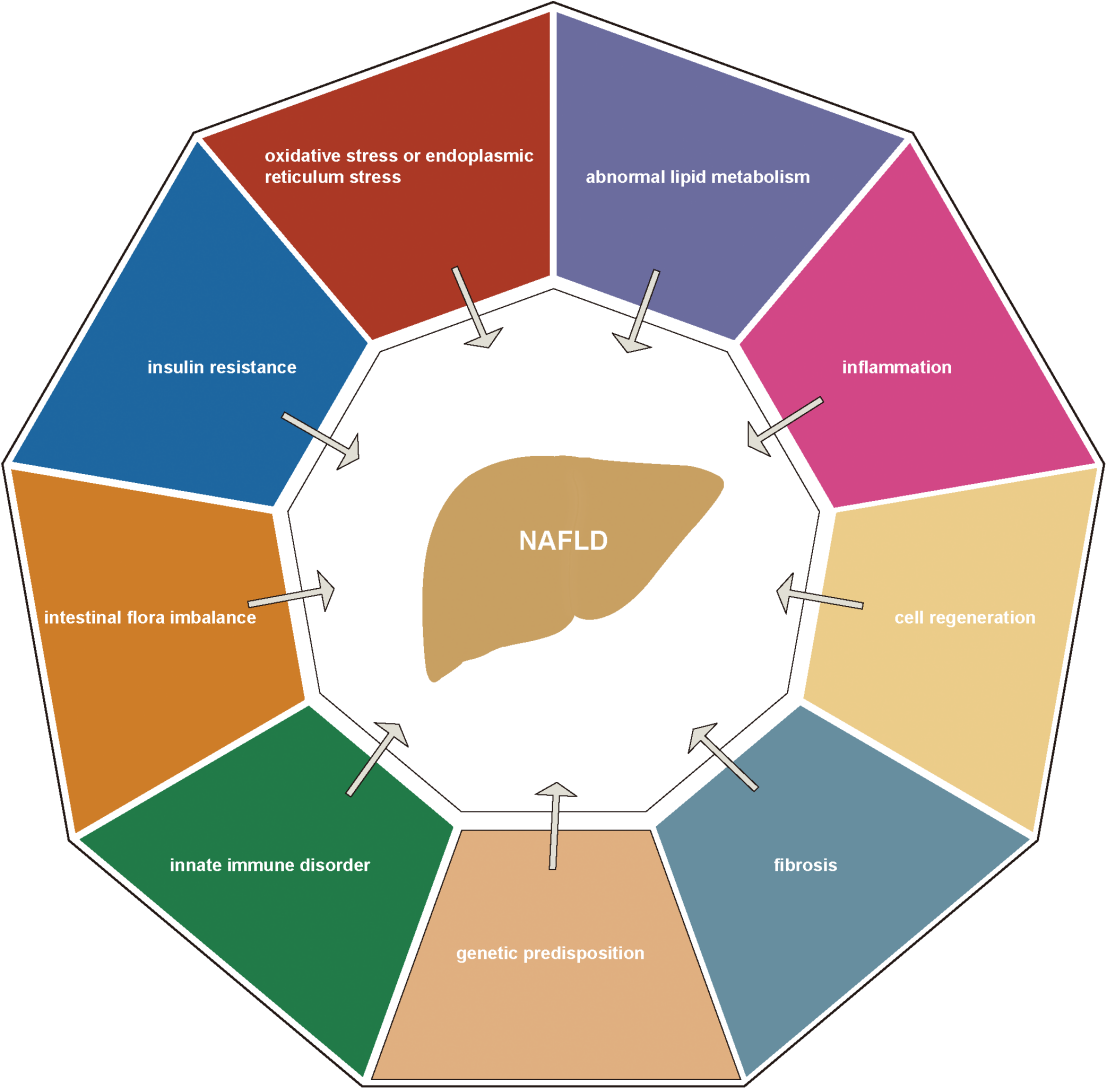
Current pathogenesis of NAFLD.

The current pathogenesis of NALFD has not yet been clarified. ‘Multiple-hit’ theory was used and widely considered as an accurate measure of NAFLD pathogenesis, including oxidative stress (OS) or endoplasmic reticulum (ER) stress, abnormal lipid metabolism, inflammation, cell regeneration, fibrosis, genetic predisposition, innate immune disorder, intestinal flora imbalance and insulin resistance (**Figure 1**). Treatment strategies currently used can have a certain therapeutic effect on specific pathogenesis but may aggravate other pathological factors that affect the prognosis of the disease. For example, current insulin therapies and insulin sensitizers may exacerbate lipid accumulation in the liver, by increasing hepatic lipid synthesis (6). To date, there are no FDA-approved drugs for the treatment of NAFLD (7, 8). Therefore, new treatment strategies need to be found to consider multiple pathological factors and targets.

Fortunately, there are a large number of studies focusing on extracts of natural plants to improve NAFLD, such as resveratrol of Reynoutria japonica Houtt. [Polygonaceae] (9), Geniposide of Gardenia jasminoides J.Ellis [Rubiaceae] (10) and Baicalin of Scutellaria baicalensis Georgi [Lamiaceae] (11). Not only that, several compositional strategies of natural plants guided by Traditional Chinese Medicine (TCM) theory such as Zhifang I Decoction (12), zaozhu yinchen Recipe (13) and Jianpi Shugan Recipe (14), have achieved some effect in clinical studies for the treatment of NAFLD. These natural plants (some including animals and ores) used under the guidance of TCM theory are defined as Chinese herbal medicines (CHM).

In TCM theory, since the disease is a dynamic process, there may be different patterns in different phases of the disease. ‘Zheng’ (TCM syndrome), has been a central diagnostic concept in TCM for thousands of years and is defined as the pattern of symptoms and signs of a patient at a particular stage of the disease dynamic process (15). Dampness-Heat Syndrome (DHS) is the most common ZHENG in TCM, highlighted by chronic low-grade systemic inflammation, which predisposes insulin resistance (IR) and causes various metabolic disorders (16). A recent clinical study revealed that DHS is the most common TCM syndrome in patients with T2DM (17).

However, the philosophical-based TCM theory lacks the elaboration of modern scientific language, which makes it difficult to directly guide the modern pharmacological and molecular mechanism research of CHM. Although a variety of studies have shown that some herbs and their active ingredients have the effect of clearing heat and removing dampness and have obvious anti-inflammatory and antioxidant effects in recent years (18, 19). The biological mechanism behind ZHENG has not been clarified, and there is a lack of connection with modern research on NAFLD, which poses an obstacle to the use of herbs under the guidance of the relationship between the characteristics of DHS and the pathological features of NAFLD

### 1.1 Perspectives of TCM on the etiology and pathogenesis of NAFLD

**Figure 2:**
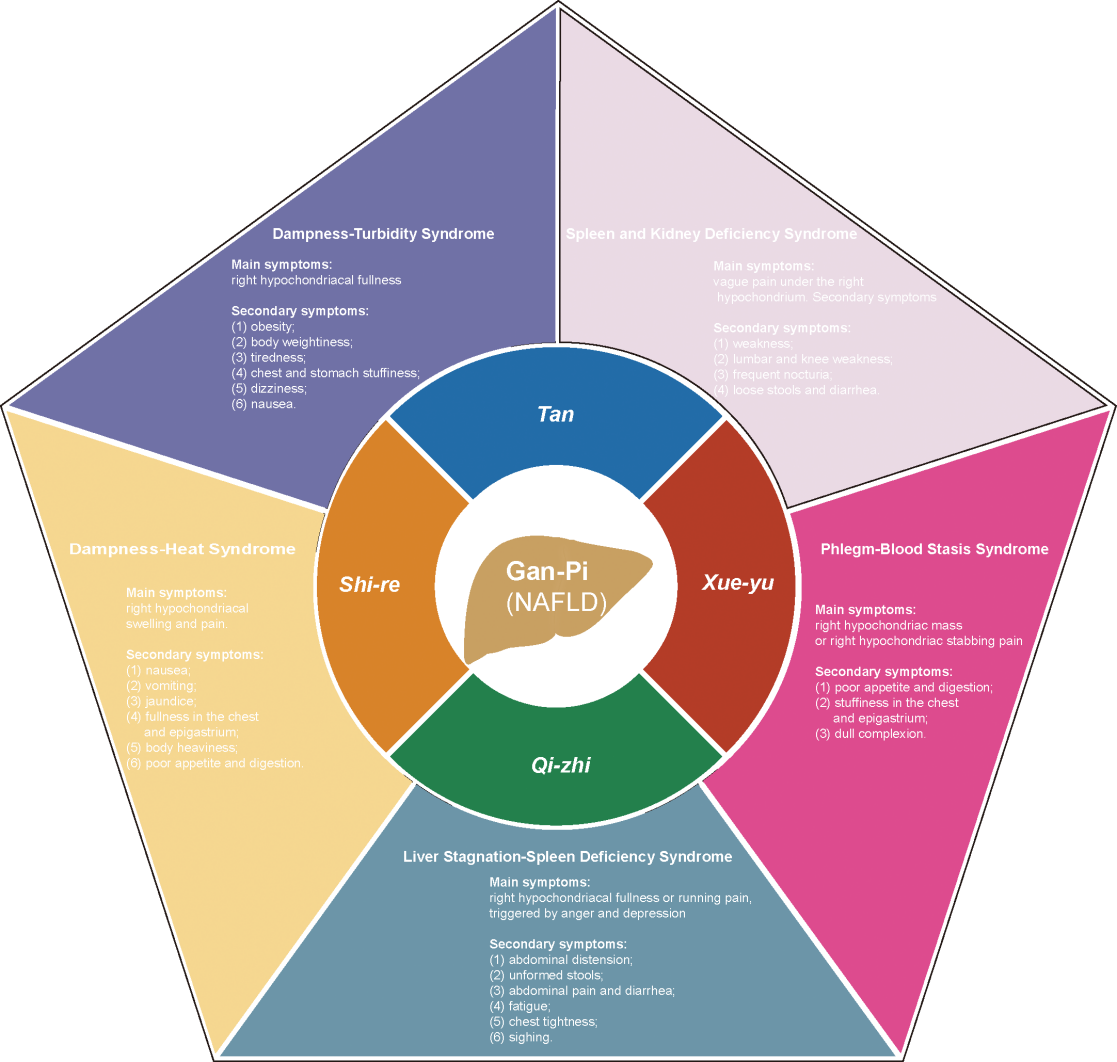
The pathological factors of Gan-Pi and the corresponding classification of symptoms of ZHENGs in TCM theory. dampness-heat (shi-re in Chinese), phlegm (tan in Chinese), blood stasis (xue-yu in Chinese) and qi stagnation (qi-zhi in Chinese).

NAFLD was discovered and named by Jurgen Ludwig in 1980 (20), and in TCM theory, there is no clear disease description for NAFLD and NASH. According to the disease characteristics of NAFLD, we can refer to the TCM theory of ‘Gan-Pi’ ‘Fei-Qi’ and ‘Ji-Ju’. A great deal of work has been done to determine the modern TCM name of NAFLD, which was finally determined as ‘Gan-Pi’ (21). In the TCM theory, the etiology of NAFLD is mainly due to dampness-heat (shi-re in Chinese), phlegm (tan in Chinese), blood stasis (xue-yu in Chinese) and qi stagnation (qi-zhi in Chinese). According to the differences in etiology factors and symptoms manifested by the disease, ZHENGs of NAFLD can be divided into DHS and other syndromes such as Dampness and Turbidity Syndrome, Liver Stagnation and Spleen Deficiency Syndrome, Phlegm and Blood Stasis Syndrome, and Spleen and Kidney Deficiency Syndrome (22). **Figure 2** details the four pathological factors that lead to Gan-Pi and the corresponding five ZHENGs classifications. In addition, the Main and secondary symptoms of the ZHENGs are elucidated. Of note, DHS is the most common syndrome related to this disease (23, 24).

### 1.2 Modern cognition and interpretation of Dampness-Heat Syndrome

The relationship between DHS and energy metabolism disorders was first described in the ancient Chinese book “Huang-Di-Nei-Jing”. The ancient Chinese realized that abnormal dietary habits could lead to disorders of energy metabolism, which coincides with the modern view of a metabolic syndrome caused by excessive energy intake. Furthermore, ‘Su Wen • Qi Bing Lun’ (25) mentioned that ‘The people enjoyed rich food and became fat, experiencing internal heat and dampness. Fat made people hot, sweet made people full.’ It is pointed out that some excessive intake of fatty or sweeter foods is the cause of DHS, while the internal heat generated by fat accumulation and the series of inflammatory reactions that may result from unhealthy foods are the cause of DHS.

DHS refers to the syndrome related to ‘dampness-heat’ in various diseases, especially digestive system diseases (26–29). Although not yet elucidated, the potential molecular basis of DHS has attracted many researchers’ attention. Recently, DHS has been implicated in a wide range of inflammatory conditions, including hepatitis (28, 30), nephritis (31), gastritis (32), arthritis (33), prostatitis (34) and ulcerative colitis (35). In clinical trials, DHS was found to be closely associated with levels of inflammatory factors. Liu et al. found a significant decrease in serum levels of inflammatory factors (IL-6, CRP and TNF-α) in patients with pelvic inflammatory disease with DHS after combined administration of Kangfuyan capsule(a proprietary Chinese medicine that clears heat and dispels dampness) and antibiotic (36). Similarly, in an RCT clinical study, serum levels of TNF-α and IFN-γ were found to be significantly higher in rheumatoid arthritis patients with DHS (33).

#### 1.3 Modern research status and challenges of DHS and NAFLD

More importantly, DHS is closely related to metabolic syndrome, especially NAFLD (22, 24). Several studies have revealed an association between the pathological manifestations of NAFLD and DHS (37–40). Zhang et al. observed that serum TG, TC and LDL-C were significantly elevated in patients with DHS (39). In addition, compared with other ZHENGs, NAFLD related to DHS has a higher level of ALT (38), this may be related to the chronic low-level systemic inflammatory response caused by the DHS.

#### 1.3.1 Modern research progress of classical formulas for the treatment of NAFLD

Traditional Chinese classical prescriptions are effective in the treatment of NAFLD (41–43). Ling-Gui-Zhu-Gan decoction, firstly found in the ancient classic “Jingui Yaolue”, is a representative formula under the principle of warming Yang and dampness removing. It consists of Smilax glabra Roxb. [Smilacaceae]/(Fu-Ling), Neolitsea cassia (L.) Kosterm. [Lauraceae]/(Gui-Zhi), Atractylodes macrocephala Koidz. [Asteraceae]/(Bai-Zhu) and Glycyrrhiza glabra L. [Fabaceae]/(Gan- Cao) (44). Previous studies have shown that Ling Gui Zhu Gan decoction significantly alleviates hepatic steatosis by down-regulated the expression of cytokine signaling 2 (44).Dai et al. revealed that Ling-Gui-Zhu-Gan decoction effectively improved insulin resistance in overweight/obese participants with NAFLD (45).

Yin-chen-hao Decoction, is a famous TCM formula and is used for the treatment of dampness-heat jaundice (46). It consists of Rheum palmatum L. [Polygonaceae]/(Da- Huang), Swertia chirayita (Roxb.) H.Karst. [Gentianaceae]/(Yin-chen) and Gardenia jasminoides J.Ellis [Rubiaceae]/(Gan-Cao). Lee et al. showed that Yin-chen-hao Decoction has a novel therapeutic approach for fatty liver progression in obesity mice by promoting senescence marker protein-30 metabolism (47). Moreover, Si Miao San is a classic formula consisting of four herbs, namely Atractylodes lancea (Thunb.) DC. [Asteraceae]/(Cang- Zhu), Phellodendron amurense Rupr. [Rutaceae]/(Huang-Bai), Achyranthes bidentata Blume [Amaranthaceae]/(Niu-Xi) and Coix lacryma-jobi L. [Poaceae]/(Yi- Yi-Ren), to clear heat and dispel dampness. Previous studies have revealed that Si Miao san could attenuate NAFLD by modulating hepatic lipid metabolism and gut microbiota (48).

#### 1.3.2 Modern research progress of Chinese patent medicine for the treatment of NAFLD

For the treatment of NAFLD related to DHS, In China, CHM compounds targeting DHS to treat NAFLD have been approved as Chinese patent medicine (CPM) and some are in clinical trials (referred to http://www.chinadrugtrials.org.cn.To 2022.04.30). Although there are some CPMs for DHS-related NAFLD in clinical practice, it is difficult to find the precise target for improving NAFLD through molecular research because of the diversity of the composition of CHM. One of the current strategies is to explore the molecular mechanisms behind the prescription by breaking it down into individual herbs or effective ingredients.

In fact, from the theory or practice of TCM, some breakthroughs have been made in exploring the active ingredients of TCM, such as the anti-malarial effect of artemisinin, an extract of Artemisia annua L. [Asteraceae] (49), and the anti-APL(acute promyelocytic leukemia) effect of arsenic trioxide, an extract of arsenic (50). Furthermore, a wide variety of pharmacological research on herbs and active ingredients has also been focused on NAFLD, although the linkage of herbs between individual studies is subtle and a review combining the theory of medication guidance of herbs with pharmacological research is lacking. Clarifying the actions and mechanisms of these herbs and active ingredients is fundamental to elucidating the molecular insights and therapeutic perspectives of TCM on NAFLD.

**Figure 3:**
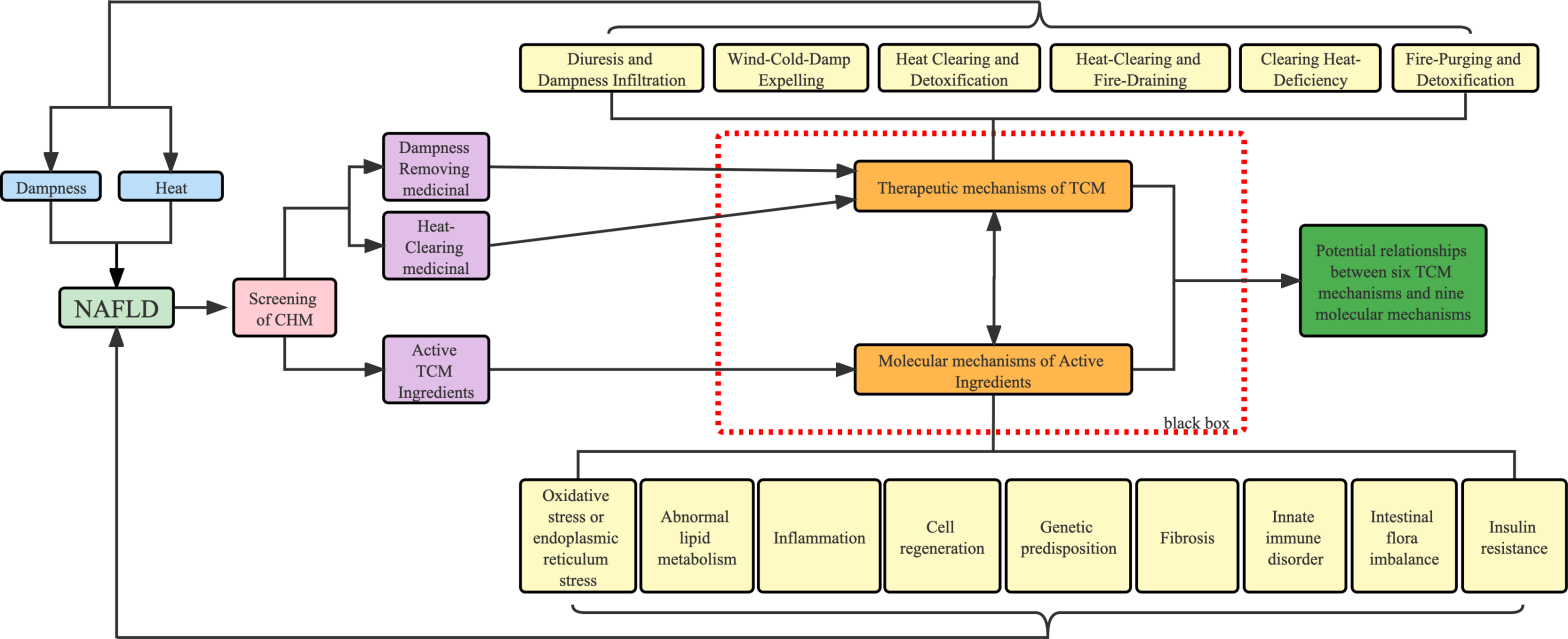
Workflow of this review. Blue represents the causative factors of NAFLD, purple represents the method of classification of drugs and light yellow represents the different pathological mechanisms.

Therefore, this paper aims to update and summarize the experimental evidence of TCM herbs and active ingredients for the treatment of NAFLD and focus on the mechanism underlying NAFLD with dampness and heat in TCM and propose potential prevention and treatment strategies using TCM theory. In our review, based on the clinical evidence of DHS and NAFLD, we updated and summarized single herbs from CPM and animal experiments that are consistent with TCM for dispelling dampness and clearing heat, and used the TCM theory of drug efficacy classification to establish the link between molecular mechanisms of herbs and their effective ingredients and TCM theory (**Figure 3**). Hopefully, it will increase the scientific profile of TCM theories and promote the modernization of TCM.

## 2 Herbs and active TCM ingredients

### 2.1 Active TCM ingredients with dampness removing effect in TCM theory

‘Dampness’ causes disturbance of circulation and hypoxia in adipose tissue and the small intestine, leading to an increase in inflammatory factors, a decrease in adiponectin, a disturbance of glucose metabolism, an imbalance of intestinal flora and an increase in lipopolysaccharide (51). In TCM theory, anti-dampness herbs can be further divided into the following: Diuresis and Dampness Infiltration (Li-Shui-Shen-Shi) Medicinal, Wind and Dampness-dispelling (Qu-Feng-Chu-Shi) Medicinal and Resolving Turbidity (Qu-Shi-Hua-Zhuo) Medicinal. In accordance with the experimental model and the specific molecular mechanism involved, we have classified and summarized the different active components (**Table 2**).

#### 2.1.1 Diuresis and dampness infiltration medicinal

##### 2.1.1.1 *Alisma plantago-aquatica subsp. orientale (Sam.) Sam. [Alismataceae]*/Ze-Xie (Chinese)

Ze-Xie is the stem tuber of Alisma plantago-aquatica subsp. orientale (Sam.) Sam. [Alismataceae]. Medicinal efficacies are the promotion of urination, purging of dampness and the discharging of heat in TCM theory. It has been reported that Ze-Xie could prevent NAFLD by improving intestinal microecology and regulating genes related to cholesterol metabolism (52). Alisol A, Alisol B and Alismatis Rhizoma Triterpenes have been proven effective on NAFLD (**Table 1**). Alisol A 24-acetate (AA-24-a) is one of the main active triterpenoid compounds isolated from Alisma plantago-aquatica subsp. orientale (Sam.) Sam. [Alismataceae]. AA-24-a can improve the abnormal accumulation of lipids in the liver by regulating a variety of molecular mechanisms, such as improving liver lipid deposition through ABCA1/ABCG1 pathway in Apoe−/− mice model (53), binding to HMG-COA reductase (54), activating PKA-mediated hormone-sensitive lipase phosphorylation and down-regulating perilipin A *in vivo* and *in vitro* (55). Alisol B 23-acetate (AB-23-a), a natural triterpenoid, can induce mobilization of internally stored calcium, leading to autophagy by activating the CAMKK-AMPK- pathway *in vitro* (56), to improve abnormal lipid metabolism and reduce cytotoxicity. Furthermore, it can improve cases of abnormal lipid metabolism, inflammation and fibrosis by activating FXR (57). Meanwhile, one study included fourteen terpenoids isolated from Alisma plantago-aquatica subsp. orientale (Sam.) Sam. [Alismataceae], has shown that it could promote peripheral IR by up-regular glut4 expression (58).

**Table 1:** Chinese patent medicine treating NAFLD target to DHS.

Compounds	Prescriptions	Register ID	Status
Da Huang Li Dan Pills	Rheum palmatum L. [Polygonaceae]/(Da-Huang),Gymnadenia orchidis Lindl./(Shou-Zhang-Shen),Phyllanthus emblica L. [Phyllanthaceae]/(Yu-Gan-Zi)	–	approved
Huazhi Rougan Granule	Artemisia capillaris Thunb. [Asteraceae]/(Yin-Chen)Senna tora (L.) Roxb. [Fabaceae]/(Jue-Ming-Zi)Rheum palmatum L. [Polygonaceae]/(Da-Huang)Alisma plantago-aquatica L. [Alismataceae]/(Ze-Xie)Polyporus umbellatus/(Zhu-Ling)Crataegus pinnatifida var. pinnatifida [Rosaceae]/(Shan-Zha)Atractylodes lancea (Thunb.) DC. [Asteraceae]/(Cang-Zhu)Atractylodes macrocephala Koidz. [Asteraceae]/(Bai-Zhu)Citrus × aurantium L. [Rutaceae]/(Chen-Pi)Trichosanthes kirilowii Maxim. [Cucurbitaceae]/(Gua-Lou)Ligustrum lucidum W.T.Aiton [Oleaceae]/Nv-Zhen-Zi)Eclipta prostrata (L.) L. [Asteraceae]/(Mo-Han-Lian)Lycium barbarum L. [Solanaceae]/(Gou-Qi-Zi)Cirsium arvense (L.) Scop. [Asteraceae]/(Xiao-Ji)Bupleurum falcatum L. [Apiaceae]/(Chai-Hu)Glycyrrhiza glabra L. [Fabaceae]/(Gan-Cao)	–	approved
Dang Fei Li Gan Capulse	Silybum marianum (L.) Gaertn. [Asteraceae]/(Shui-Fei-Ji), Swertia pseudochinensis H.Hara [Gentianaceae]/(Dang-Yao)	–	approved
Shu gan zhi Tables	Bupleurum falcatum L. [Apiaceae]/(Chai-Hu)Citrus × aurantium L. [Rutaceae]/(Zhi-Qiao)Curcuma aromatica Salisb. [Zingiberaceae]/(E-Zhu)Seaweed/(Hai-Zao)Atractylodes lancea (Thunb.) DC. [Asteraceae]/(Cang-Zhu)Tuckahoe/(Fu-Ling)Atractylodes macrocephala Koidz. [Asteraceae]/(Bai-Zhu)Carthamus tinctorius L. [Asteraceae]/(Hong-Hua)Panax notoginseng (Burkill) F.H.Chen [Araliaceae]/(San-Qi)Crataegus pinnatifida var. pinnatifida [Rosaceae]/(Shan-Zha)Astragalus mongholicus Bunge [Fabaceae]/(Huang-Qi)Sargentodoxa cuneata (Oliv.) Rehder & E.H.Wilson [Lardizabalaceae]/(Da-Xue-Teng)	CTR20180031	IIa
Dan Shao Gan Kang Granules	Bupleurum falcatum L. [Apiaceae]/(Chai-Hu)Astragalus mongholicus Bunge [Fabaceae]/(Huang-Qi)Angelica sinensis (Oliv.) Diels [Apiaceae]/(Dang-Gui)Paeonia lactiflora Pall. [Paeoniaceae]/(Bai-Shao)Salvia miltiorrhiza Bunge [Lamiaceae]/(Dan-Shen)Schisandra chinensis (Turcz.) Baill. [Schisandraceae]/(Wu-Wei-Zi)	CTR20140038	IIb

##### 2.1.1.2 *Nelumbo nucifera Gaertn. [Nelumbonaceae]*/He-Ye (Chinese)

Nuciferine, an active alkaloid, is derived from Nelumbo nucifera Gaertn. [Nelumbonaceae], has been demonstrated to regulate FFA infiltration, inflammation and oxidative stress in an HFD-induced rat model (59). In addition, it seems that nuciferine could also limit abnormal fat accumulation by decreasing the expression of Per-Arnt-Sim Kinase (PASK) (60) and improving glycerophospholipid, linoleic acid, alpha-linolenic acid, arginine, and proline metabolism pathways (61).

##### 2.1.1.3 *Trigonella foenum-graecum L. [Fabaceae]*/Hu-Lu-Ba (Chinese)

Diosgenin (DSG), an active sapogenin component isolated from Trigonella foenum-graecum L. [Fabaceae]/ (Hu-Lu-Ba in Chinese), is used to treat diabetes nowadays (62). It has been demonstrated that diosgenin can ameliorate abnormal fat accumulation and inflammation by activating AMPK/ACC/CPT-1a and inhibiting SREBP-1C/FAS signaling pathway (63).

##### 2.1.1.4 *Reynoutria japonica Houtt. [Polygonaceae]*/Hu-Zhang (Chinese)

**Figure 4:**
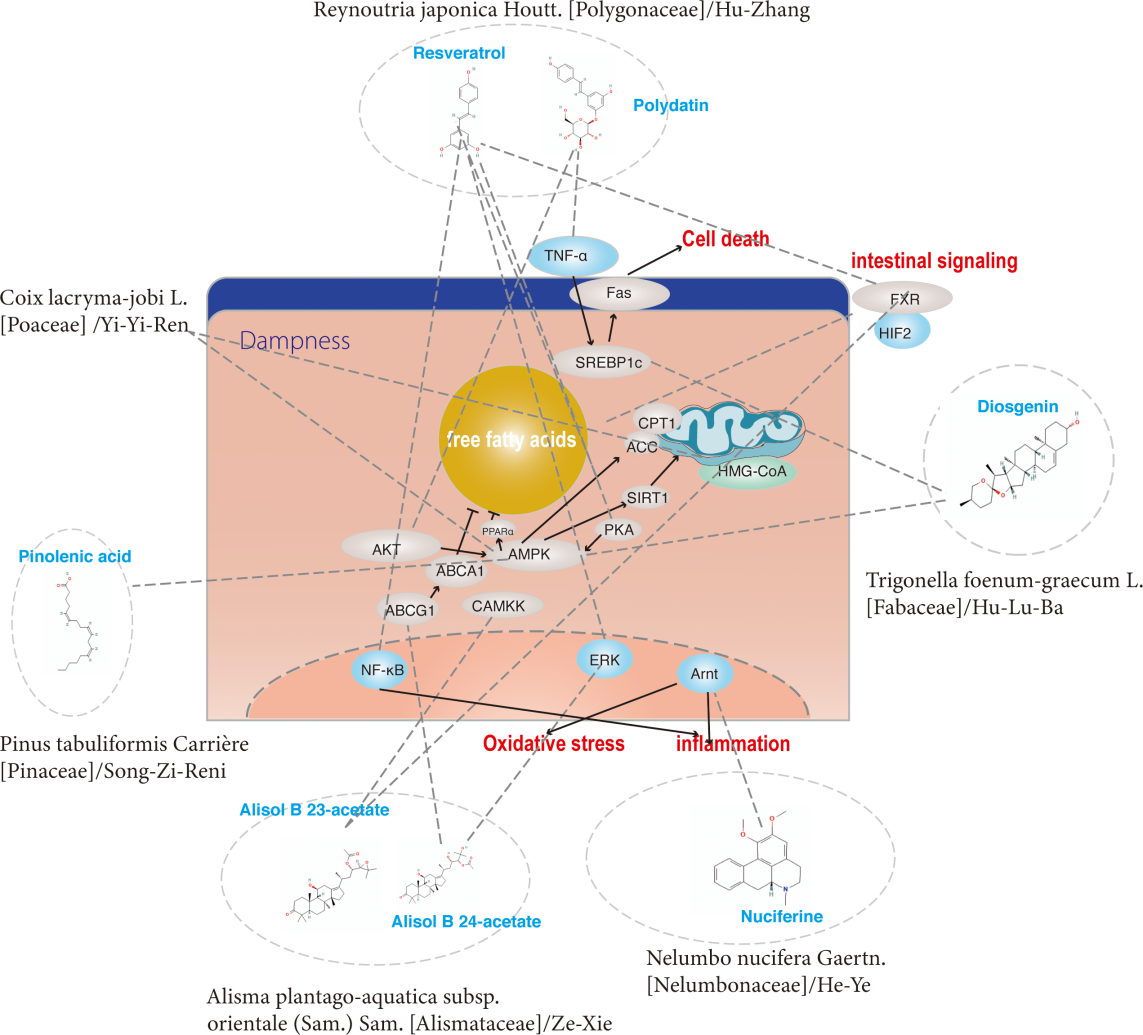
The regulatory effects on pathways induced by a group of ingredients from dispelling dampness (Qu Shi) and dissolving turbidity (Hua Zhuo) herbs.

Hu-Zhang is the root stalks and roots of Reynoutria japonica Houtt. [Polygonaceae], pertaining to Polygonum. Historically, it was used to treat dampness-heat jaundice in TCM theory. The active ingredients include resveratrol, emodin of anthraquinones, quercetin, polydatin and its derivatives of flavanols, coumarin and lignan (59). Resveratrol, pterostilbene and polydatin are the main active components and work together to exert a therapeutic effect on NAFLD (**Figure 4**). Resveratrol is a polyphenol that is derived from Reynoutria japonica Houtt. [Polygonaceae], is well known for its beneficial health properties, such as the limitation of abnormal lipid accumulation (24–28), the reduction of oxidative and ER stress (24, 26), the improvement of genetic predisposition (8) and the balance of intestinal flora (28–30). Resveratrol limited the intake and synthesis of lipids and reduced oxidative stress in HepG2 cells incubated with oleic acid and palmitic acid (24). Resveratrol also limited triglyceride accumulation *in vivo* and *in vitro*, due to a decrease in the methylation level of nrf2 promoter (8). *In vivo* studies (in rats fed with HFD) showed that resveratrol significantly increased the activity of glucose and lipid metabolism and decreased rats’ behavioral and cognitive impairment (25). Nevertheless, recent studies have shown that resveratrol increases autophagy by stimulating fatty acid β-oxidation in cells *via* the cAMP-PRKA-AMPK-SIRT1 signaling pathway (31). In addition, in an *in-vitro* study, induced by high glucose, resveratrol was shown to limit lipogenesis and enhance mitochondrial activity (27). Resveratrol also improved insulin sensitivity and lipid metabolism by increasing the abundance of intestinal-specific bacteria *in vivo* (30). Furthermore, recent studies have clarified that resveratrol induced gut barrier impairment, by inhibiting colonic CB1 and abrogated the aggravated intestinal inflammation *via* activating CB2 in C57bl/6J mice fed HFD models (29). Subsequently, increasing evidence has demonstrated the role of Resveratrol in regulating not only the intestinal microenvironment (30) but also the progress of inflammation (32, 33). Resveratrol was also shown to regulate the expression of key molecules related to SIRT1, LXR, FXR of regulating autophagy (34) and GPAT-1 DGAt2 of PKC membranous translocation (35) in HFD-induced rat models. Pterostilbene is a methoxylated derivative of resveratrol and a recent study has demonstrated that pterostilbene had higher antioxidant and anti-inflammatory properties compared with resveratrol in Wistar rats, induced by high-fat high-fructose models (36). In addition, it was reported that the compound A19, a resveratrol-curcumin hybrid reduced the inflammatory response and decreased PA-induced ERK phosphorylation *in vivo* and *in vitro* (37). Recent studies showed that Reynoutria japonica Houtt. [Polygonaceae] 80% EtOH extract (POCU1b) could decrease lipid accumulation, reduce inflammation and IR by activating pancreatic lipase, CAMP-dependent PDE activity, AMPK activation, and SOCS-3 suppression in rats fed with HFD (38). Polydatin is a stilbenoid compound derived from the rhizome of Reynoutria japonica Houtt. [Polygonaceae]. One of the main properties of polydatin is its hepatoprotective activity by reducing liver lipid accumulation, inflammation (39), insulin resistance (40) and apoptosis (41). It has also been demonstrated that polydatin suppresses the expression of TNF-α and SREBP-1c in SD rats fed HFD models (39). In addition, it has been revealed that treatment with polydatin significantly decreased the transcription factor TFEB and subsequently restored the lysosomal clearance of autophagosomes (41). Recently, a study demonstrated that polydatin could regulate ubstrate 2 expression levels and AKT phosphorylation to prevent insulin resistance in an HFD-induced rat model (40).

##### 2.1.1.5 *Coix lacryma-jobi L. [Poaceae]/*Yi-Yi-Ren (Chinese)

Coix lacryma-jobi L. [Poaceae] has served as nourishing food and used in TCM for many years for the treatment of inflammatory diseases. Several studies have demonstrated that Coix lacryma-jobi L. [Poaceae] could inhibit abnormal lipid accumulation (64, 65). A recent *in vivo* study has demonstrated that the ethanolic extract of adlay seeds has efficacy in the inhibition of lipogenesis and induction of fatty acid β oxidation in the liver (64). In addition, Coix lacryma-jobi L. [Poaceae] oil reduced fat accumulation by inhibiting the *p*-AMPK/sepp1/apoer2 pathway (65).

#### 2.1.2 Wind-cold-damp expelling medicinal

Wind-Cold-Damp Expelling Medicinal has the efficacy of expelling wind and removing dampness, relieving pain. Amongst these, Papaya and Pine Nuts (Pinus Koraiensis) were reported to improve NAFLD.

##### 2.1.2.1 *Chaenomeles lagenaria (Loisel.) Koidz. [Rosaceae]/*Mu-Gua (Chinese)

Mu-Gua is the nearly mature fruit of Chaenomeles lagenaria (Loisel.) Koidz. [Rosaceae], pertaining to Rosaceae. Almost all parts of Chaenomeles lagenaria (Loisel.) Koidz. [Rosaceae] can be used, especially the fruit. It is a nutritional source that is high in fibre, minerals and strong antioxidants including vitamins A, C and E. A recent *in vivo* study revealed that Chaenomeles lagenaria (Loisel.) Koidz. [Rosaceae] reduces the accumulation of lipids in the liver, inhibits the lipogenic pathway, improves the balance of antioxidation and reduces inflammation (66).

##### 2.1.2.2 *Pinus tabuliformis Carrière [Pinaceae]/*Song-Zi-Ren (Chinese)

Pinolenic acid (all-cis-5, 9, 12–18:3), a polyunsaturated fatty acid, has been isolated from pine nut oil in Pinus tabuliformis Carrière [Pinaceae]. Pinus tabuliformis Carrière [Pinaceae] Oil has historically been a dietary supplement for preventing obesity and metabolic dysregulation (67, 68). A study demonstrated that pinolenic acid could improve cases of lipogenesis and oxidative stress by regulating AMPK/SIRT1 signaling pathway (67). An *in vitro* study revealed that pinolenic acid down-regulated the lipid anabolic pathway in HepG2 cells by reducing the expression of genes related to lipid synthesis and lipoprotein uptake (68).

### 2.2 Active TCM ingredients with heat-clearing effect in TCM theory

#### 2.2.1 Heat clearing and detoxification medicinal

##### 2.2.1.1 *Andrographis paniculata (Burm.f.) Nees [Acanthaceae]*/Chuan-Xin-Lian (Chinese)

Andrographis paniculata (Burm.f.) Nees [Acanthaceae], a herb used in Chinese, Indian and Thai remedies that are commonly used to treat infections, colds, and diarrhea. Terpenoids are the most attractive phytonutrients of Andrographis paniculata (Burm.f.) Nees [Acanthaceae] and several diterpenoids have also been identified, including andrographolide (AND), 14-deoxy-11,12-didehydroandrographolide (deAND), isondrographolide, 14-acetylandrographolide, and 14-deoxyandropholide. These diterpenoids have been reported to have many biological functions, including anti-oxidative stress, anti-inflammation, anti-apoptosis and regulation of lipid metabolism (69, 70). A large number of clinical researchers reported the anti-inflammatory and hepatoprotective effects of andrographolide (AND) (71–73). deAND could reduce cholesterol accumulation by suppressing the expression of NLRP3 and caspase-1 and also reduce HFHC diet-induced apoptosis by lowering the caspase 3/pro-caspase 3 ratios (69). Furthermore, isoandrographolide (IAN), one of the derivatives of AND, has shown improved efficacy on hyperlipidemia and fat accumulation in the liver and showed comparatively higher success with lower irritability and more stability than AND (70).

##### 2.2.1.2 Bear Bile/Xiong-Dan (Chinese)

Ursodeoxycholic acid (UDCA), first identified in bear bile, has been widely used for the treatment of cholestatic liver diseases (74). Previous studies found that UDCA protected the liver from NAFLD and oxidative stress, which may be mediated by autophagy and apoptosis (75, 76). In a recent study, the expression levels of AKT/mTOR/SREBP-1 signaling pathway-related proteins were regulated by ursodeoxycholic acid (UDCA). UDCA treatment suppressed the activation of AKT, mTOR, and CRTC2 and the expression of nSREBP-1 *in vitro* (76). Additionally, it has been reported that UDCA administration promotes autophagy by activating the AMPK pathway to inhibit apoptosis (55).

##### 2.2.1.3 *Gardenia jasminoides J.Ellis [Rubiaceae]*/Zhi-Zi (Chinese)

The fruit of Gardenia Gardenia jasminoides J.Ellis [Rubiaceae] is a herb for cleaning away toxicity in TCM. Recent studies showed that the extract of Gardenia jasminoides J.Ellis [Rubiaceae] reduced inflammation by suppression of JNK2/1 signaling pathways (77) and the expression of cytokines such as TNF-α and IL-6 in adipose tissue (78). Moreover, active ingredients of Gardenia jasminoides J.Ellis [Rubiaceae], such as genipin and geniposide, have an alleviating effect on fatty liver in HFD-induced rat models (79, 80). Geniposide has many biological effects, such as anti-inflammation, regulation of the amount of intestinal flora and limitation of abnormal lipid accumulation (10, 81). It has been reported that geniposide enhanced the reduction of antioxidative stress and inflammation by up-regulating the protein expression of Nrf2/HO-1 and AMPK signaling pathway *in vivo* and *vitro* (10). Furthermore, recent *in vivo* studies revealed that the ability to suppress intracellular lipid accumulation may be due to increasing the expression of PPARa (82). Subsequently, geniposide was found to reduce the signaling of gut-derived lipopolysaccharide (LPS), protecting the gut barrier function by down-regulating the RHOA/ROCK signaling pathway (81). Geniposide also improved hepatic free fatty acid metabolism in rats induced by a high-fat diet by regulating the AMPK–Malonyl-CoA-FFA axis (80).

Genipin is the aglycone derived from geniposide, the most abundant iridoid glucoside constituent of Gardenia jasminoides J.Ellis [Rubiaceae]. Subsequently, it has been reported that genipin inhibited hepatic oxidative stress and prevented mitochondrial dysfunction in Aging SD rat models and palmitate-treated L02 cell models (83). In addition, pyroptosis is a highly inflammatory form of lytic programmed cell death that occurs most frequently upon infection with intracellular pathogens and likely forms part of the antimicrobial response. Genipin could inhibit UCP2-mediated pyroptosis to reverse HFD-induced liver damage (79).

#### 2.2.2 Heat-clearing and fire-draining medicinal

##### *Senna tora (L.) Roxb. [Fabaceae]/*Jue-Ming-Zi (Chinese)

Senna tora (L.) Roxb. [Fabaceae] is an annual herb that grows in tropical countries in Asia, which is widely cultivated in China and usually used to treat insomnia. Meng et al (84), reported that Senna tora (L.) Roxb. [Fabaceae] also could alleviate NAFLD in rat models, which can significantly reduce the levels of TNF-α, IL-6, IL-8 and MDA in the liver and serum.

##### 2.2.2.1 Scutellaria baicalensis Georgi [Lamiaceae]/Huang-Qin (Chinese)

The dried root of Scutellaria baicalensis Georgi [Lamiaceae] belonging to Lamiaceae, is a herb used for relieving heat, (fire-draining). Scutellaria baicalensis Georgi [Lamiaceae] extract (SBE) exerted regulating effects on hyperglycemia, hypertriglyceride, and hypercholesterolemia (85). Baicalin, the major flavonoid in Scutellaria baicalensis Georgi [Lamiaceae], has been demonstrated to have anti-lipotoxicity, through the regulation of AMPK-mediated SREBP signaling pathway (86). Baicalin was also reported to attenuate pyroptosis by inhibiting NLRP3– GSDMD signaling *in vitro* of HepG2 cells (11).Baicalein is a flavonoid found in Scutellaria baicalensis Georgi [Lamiaceae], which has been usually used for the treatment of fever, viral infections, bacterial infections, inflammation, and cancer (87, 88). Baicalein induced apoptosis and autophagy of breast cancer cells *via* inhibiting the PI3K/AKT signaling pathway *in vivo* and *vitro* (88). In previous studies, baicalein was also reported to reduce oxidative stress and abnormal lipid metabolism by activating AMPK and suppressing SREBP1 cleavage in oleic acid-induced HepG2 cells and HFD-induced mice models (89). It also was revealed that it attenuated lipid metabolism, inflammation and fibrosis in mice by suppressing key regulators such as SREBP-1c, FASN, PPARα and Col1A1 (90).

##### 2.2.2.2 *Sophora flavescens Aiton [Fabaceae]/*Ku-Shen (Chinese)

Sophora flavescens Aiton [Fabaceae] (Ku-Shen) is a monarch herb in TCM used for the treatment of DHS which has achieved high efficacy in treating metabolic disease in clinical practice and effect studies (91). Ku-Shen mainly contains the components oxymatrine and matrine. Oxymatrine is one of many quinolizidine alkaloid compounds extracted from the root of Sophora flavescens Aiton [Fabaceae] (Ku-Shen). It is very similar in structure to matrine, which has one less oxygen atom and has been revealed to possess various pharmacological effects, including anti-hepatitis virus infection (92), anti-hepatic lipid abnormal accumulation and anti-inflammation (93). A recent study has reported that oxymatrine regulates lipid accumulation in the liver by increasing the mRNA and protein levels of PPARα, CPT1 and MTTP in fatty liver rats (93). Matrine is also originally isolated from the plant Sophora Flavescens and it has been used as an anti-inflammation drug in China (94). Matrine reduced ER stress and mitochondrial dysfunction *via* SERCA pathway in HFD-fed mice (95). Additionally, matrine treatment enhanced HSP72 and down-regulated mTOR to reduce inflammation and fibrosis fat accumulation in C57BL/6J mice fed MCD models (96).

#### 2.2.3 Clearing heat-deficiency medicinal

##### 2.2.3.1 *Artemisia annua L. [Asteraceae]/*Qing-Hao (Chinese)

Artemisia annua L. [Asteraceae], belongs to the Asteraceae family and grows wild in Asia (97). It has been used for the treatment of various diseases in China (98). Many studies had reported its anti-inflammation efficacy (99, 100). Artemisinin, a sesquiterpene lactone, inhibits the activity of *Plasmodium Falciparum* and other malarial parasites. A recent study revealed a water extract of Artemisia annua L. [Asteraceae] could regulate lipid accumulation and oxidative stress in HepG2 cells and high-fat diet-fed mice, as well as weight gain and liver damage *in vivo* (101).

#### 2.2.4 Fire-purging and detoxification medicinal

##### 2.2.4.1 *Rheum palmatum L. [Polygonaceae]/*Da-Huang (Chinese)

Rheum palmatum L. [Polygonaceae] is a species of flowering plant in the knotweed family Polygonaceae which has been used for fever-associated diseases (102). Rhein (4,5-dihydroxyanthraquinone-2-carboxylic acid) is an anthraquinone and one of the major components of Rheum palmatum L. [Polygonaceae]. Some studies have shown that rhein enhances lipid metabolism in animals (103) and inhibits cell proliferation, inflammation and apoptosis (104). Furthermore, a recent study showed that rhein activated the UCP1 gene by antagonizing the repressive effect of LXR on UCP1 expression to improve lipid metabolism *in vivo* and vitro (103). Interestingly, rhein has been shown to have immunoregulatory functions. It has been reported to regulate lipogenesis through LXR-mediated SREBP-1c and shift the imbalanced Th1/Th2 response in the liver by modulation of cytokine signaling (105). Furthermore, rhein lysinate (RHL), which is the salt of lysine and rhein, protects the liver in mice from oxidative stress injury and inflammation by decreasing the expression of TNF- α, IL-6, NF- κB, SREBP-1c and Fas (106). Emodin (1,3,8-trihydroxy-6-methylanthraquinone), one of the major bioactive hydroxyanthraquinone in the root and rhizome of Rheum palmatum L. [Polygonaceae], has been shown to have anti-inflammatory, antioxidative and hepatoprotective effects (107, 108). Some studies have suggested that emodin is effective in reducing lipid accumulation in rats (109, 110). Moreover, emodin has effects on NAFLD caused by a high fed diet and has been reported to improve lipid accumulation *via* the ERS–SREBP1c pathway (110). Some studies have suggested that emodin is effective in reducing hepatic lipogenesis by regulating of AMPK signaling pathway in Zebrafish fed Egg yolk powder models (111). Furthermore, recent research demonstrated that emodin alleviated hepatic lipid accumulation by inhibiting SREBP1 activity *via* the camkk-AMPK-mTOR-p70s6k signaling pathway (109) and promoting the activity of AMPK and decreasing the gene expression of the biosynthesis of fatty acids and TG (112).

##### 2.2.4.2 *Aloe vera* (*L.*) *Burm.f*. [*Asphodelaceae*] /Lu-Hui (Chinese)

Aloe vera (L.) Burm.f. [Asphodelaceae] is a species of plant belonging to the genus Aloe, and has been recorded as one of the ten most frequently used herbs for constipation (113). Aloin, also known as barbaloin, is a natural aloe- derived anthraquinone compound that can ameliorate oxidative stress, anti-apoptosis and anti-inflammation on liver diseases (114, 115). A recent study also revealed that aloin can enhance antioxidant, anti-inflammatory and anti-apoptotic activity by activating Nrf2/HO-1 signaling in Nrf2 KO (Nrf2−/−) C57BL/6 mice model fed CDAAH diet models (116). Moreover, aloin could reduce oxidative stress, liver inflammation in rats fed HFHFD (18).

## 3 Conclusions and perspectives

Guided by a relevant and diverse philosophical system that emphasizes holistic health balance, TCM has become an important raw material for the development of new anti-NAFLD drugs. DHS is a concept in TCM, a general term for a series of symptoms summarized by ancient Eastern medicine (117). TCM theory gradually developed a method of using herbs to treat this symptom in continuous attempts, and to establish a relationship between the composition of different herbs and different symptoms. Obviously, the theory has certain limitations, such as unknown pharmacological effects, lack of clinical experimental evidence, etc. However, due to the special way of using herbs, usually a combination of multiple herbs, is an obstacle to finding specific, effective ingredients. Fortunately, an ever-increasing number of herbs are being investigated *via* well-designed experiments using various methods of isolating active ingredients and different types of animal models, to provide evidence in support of beneficial effects in liver disease. In this review, to benefit the discovery of herb-derived anti-NAFLD drugs, Traditional Chinese medicine for the treatment of damp-heat syndrome and its effective components are discussed.

Hypoxia and inflammation are important features of DHS, which are common in chronic low-grade systemic inflammation with IR, which eventually leads to free fatty acids metabolism disorder (51). Non-liver organs and non-liver cells such as gut, adipose, skeletal muscle, brain and immune systems contribute to NAFLD progression in the liver. When the free fatty acids disposal pathways of over-loaded fatty acids are over-occupied, fatty acids then form lipotoxic species that could cause cell death, oxidative stress, ER stress and inflammation, and activate hepatic stellate cells (HSC) and cause liver fibrosis.

In TCM theory, Alisma plantago-aquatica subsp. orientale (Sam.) Sam. [Alismataceae]/Ze-Xie, Nelumbo nucifera Gaertn. [Nelumbonaceae]/He-Ye, Trigonella foenum-graecum L. [Fabaceae]/Hu-Lu-Ba, Reynoutria japonica Houtt. [Polygonaceae]/Hu-Zhang, Coix lacryma-jobi L. [Poaceae]/Yi-Yi-Ren, Chaenomeles lagenaria (Loisel.) Koidz. [Rosaceae]/Mu-Gua, and Pinus tabuliformis Carrière [Pinaceae]/Song-Zi-Ren could clear dampness and have the effect of anti-NAFLD, that is currently reported in the NAFLD or the NASH model (**Figures 4**, **6A**). Furthermore, each herb could ameliorate NAFLD with multiple targets and mechanisms, such as (**Tables 2**, **3**). Specifically, Alisol B 23-acetate, Nuciferine, Diosgenin, Resveratrol and Pterostilbene were proven reducers of oxidative and ER stress in NAFLD models. In addition, Alisol B 23-acetate, Alisol A 24-acetate, Nuciferine, Diosgenin, Resveratrol, Pocu1b, Polydatin, Pinus tabuliformis Carrière [Pinaceae] oil and Pinolenic acid could improve NAFLD by reducing abnormal lipid accumulation in the liver. Furthermore, Alisol B 23-acetate, Nuciferine, Diosgenin, Resveratrol, Pterostilbene, Polydatin, Resveratrol-curcumin hybrid, a19 and Pocu1b were proven to alleviate liver inflammation in animal models. Moreover, Alisol B 23-acetate, Resveratrol and Polydatin could affect hepatocyte regeneration through different mechanisms. Resveratrol is one of the most reported effective components of traditional Chinese medicine. In the current research, the specific mechanism of its regulation of NAFLD may be related to oxidative ER stress, abnormal lipid metabolism, inflammation, fibrosis, genetic predisposition, intestinal flora imbalance and insulin resistance.

**Table 2:** Main mechanisms of CHM treating Congestion of Dampness Turbidity of NAFLD and its complications by dispelling dampness (Qu Shi) and dissolving turbidity (Hua Zhuo).

Active ingredients	Source (English name/pinyin)	Efficacy of TCM theory	Experimental model	Molecule Mechanisms	Pathological mechanism	Ref.
Alisol B 23-acetate	Alisma plantago-aquatica subsp. orientale (Sam.) Sam. [Alismataceae]/Ze-Xie	Diuresis and dampness infiltration	Hp3b,HepG2,Hela,Sk-br-3,Mda-MB-231,MCF-7,Pc3,C666-1 cell	Induces mobilization of internally stored calcium, leading to autophagy by activating the CAMKK-AMPK- pathway	2,4	(56)
			C57bl/6 mice MCD model	Activating farnesol X receptor	1,3,5	(57)
Alisol A 24-acetate			Apoe−/− mice model	Alisol A24-acetate improved liver lipid deposition through ABCA1/ABCG1 pathway.	2	(53)
			T3-l1 mouse fibroblast cell model	Activation of PKA-mediated hormone-sensitive lipase phosphorylation and ERK-mediated down-regulation of perilipin A	2	(55)
			ICR mice lipid emulsion-induced model	Binding to 3-hydroxy-3-methylglutary-coenzyme A (HMG-CoA) reductase,	2	(54)
Alismatis rhizoma triterpenes			C57bl/6j mice HFD model";"C2C12 cells model	Promoting IR by elevated GLUT4 expression	6,9	(58)
Methanol extract of the tuber of Alisma plantago-aquatica subsp. orientale (Sam.) Sam. [Alismataceae]			Tunicamycin-induced and HFD induced C57bl/6 mice model";"tunicamycin-treated and pa-treated HepG2 cell	Inhibition of expression of the hepatic lipogenic genes and VLDLR, and enhancement of Apoe secretion to improve ER stress	1	(118)
Alisma plantago-aquatica subsp. orientale (Sam.) Sam. [Alismataceae]extract			Sprague dawley (SD) rats HFD model	Altering intestinal microecology and regulating genes related to cholesterol metabolism	2,8	(52)
Nuciferine	Nelumbo nucifera Gaertn. [Nelumbonaceae]/He-Ye		Oleic acid (OA)-induced HepG2 cells	Regulate Per-Arnt-Sim kinase expression	1,2,3	(60)
			golden Syrian hamsters fed HFD model	Regulate FFA infiltration, inflammation and oxidative stress	1,2,3	(59)
			SD rats fed HFD model	Improve glycerophospholipid, linoleic acid, alpha-linolenic acid, arginine, and proline metabolism pathways	2	(61)
Diosgenin	Trigonella foenum-graecum L. [Fabaceae]/Hu-Lu-Ba		L02 cells incubated with palmitic acid	By activating AMPK/ACC/CPT-1A and inhibiting SREBP-1c/FAS signaling pathway	2,3	(63)
Resveratrol	Reynoutria japonica Houtt. [Polygonaceae]/Hu-Zhang		HepG2 cells. Incubated with oleic acid and palmitic acid	Limit the intake and synthesis of lipids and reduce oxidative stress.	1,2	(119)
			HFD fed C57/bl6 mice model";"HepG2 cells treated with d-glucose model	Reduce the methylation level of Nrf2 promoter	2,6	(9)
			HFD fed SD rats model	Improve liver and glucose and lipid metabolism disorders, improve behavioral and cognitive impairment	2	(120)
			PA-induced HepG2 cell	Improve lipid metabolism and redox homeostasis and oxidative stress by activating the PKA/AMPK/PPARα signaling pathway	1,2	(121)
			129/svj mice induced by HFD diet model";"HepG2 cells treated with PA	Stimulate fatty acid β-oxidation by inducing autophagy through the camp-Pka-Ampk-Sirt1 signaling pathway	4	(122)
			HepG2 cells induced by high glucose	Limit lipogenesis and enhance mitochondrial activity	2	(123)
			SD rats fed HFD model	Induce gut barrier impairment by inhibiting colonic CB1, and abrogates the aggravated intestinal inflammation *via* activating CB2, resulting in LPS translocation suppression	8	(124)
			C57bl/6 j mice fed HFD model	Improve the intestinal microenvironment, including gut barrier function and gut microbiota composition.	8	(125)
			C57bl/6j mice fed HFD model	Improve insulin sensitivity and lipid metabolism by increasing the abundance of intestinal specific bacteria	2,8	(126)
			Wistar rats fed HFD model	Improve inflammatory oxidative stress	1,3	(127)
			C57bl/6 mice were treated with MCD model aml12 cells were treated with MCD model	Attenuate hepatic steatosis and inflammation in MCD-induced NASH by regulating autophagy	1,3	(128)
			Wistar rats fed HFD model	Down-regulate GPAT-1 and Dgat2 expression and inhibit PKC membranous translocation	9	(129)
			Wistar rats fed HFD model	Improve liver function by up regulating the expression of SIRT1, LXR and FXR	6	(130)
			Ulk1+/- mice fed with HFD model	Regulation of autophagy and NF- kappa B activity	3, 4	(131)
Pterostilbene			Wistar rats induced by high-fat high-fructose	Higher antioxidant and anti-inflammatory activities	1,3	(132)
Resveratrol-curcumin hybrid, a19			C57bl/6 mice fed with HFD model";" mouse primary peritoneal macrophage and HepG2 cell treated with PA	Down-regulated the inflammatory response";"reduce PA-induced ERK phosphorylation	3	(133)
Pocu1b			SD rats fed HFD model	Inhibitions of pancreatic lipase, cAMP-dependent PDE activity, AMPK activation, and SOCS-3 suppression	2,3,9	(134)
Polydatin			SD rats fed HFD model	Inhibiting the expression of TNF-α and srebp-1c	2,3	(135)
			SD rats fed HFD model	Improved insulin receptor substrate 2 expression levels and AKT phosphorylation	9	(136)
			C57bl/ksj-db-/db (db/db) Mice fed HFD model	Up regulating the transcription factor TFEB and subsequently restoring the lysosomal clearance of autophagosomes.	4	(137)
The ethanolic extract of adlay seeds	Coix lacryma-jobi L. [Poaceae]/Yi-Yi-Ren		C57bl/6j mice fed HFD model	Inhibition of lipogenesis and induction of fatty acid β oxidation in liver	2	(64)
Coix lacryma-jboi seed oil			Wistar rats fed HFD model	Inhibiting the p-AMPK/SEPP1/APOER2 pathway	2	(65)
–	Chaenomeles lagenaria (Loisel.) Koidz. [Rosaceae]/Mu-Gua	Wind-cold-damp Expelling	Sprague-dawley rats fed HFD model	Reduce the accumulation of lipids in the liver, inhibit the lipogenic pathway, improve the balance of antioxidation and reduce inflammation	2,3	(66)
Pinolenic acid	Pinus tabuliformis Carrière [Pinaceae]/Song-Zi-Ren		Oleic acid (OA)-induced L02 cells and HepG2 cell model	Improving lipogenesis and oxidative stress by regulating AMPK/SIRT1 signaling pathway	2,3	(67)

The current pathological mechanism of NAFLD:1.oxidative stress (OS) or ER stress, 2.abnormal lipid metabolism, 3. inflammation, 4.cell regeneration, 5.fibrosis, 6.genetic predisposition, 7.innate immune disorder, 8.intestinal flora imbalance 9. insulin resistance.

**Table 3:** Main mechanisms of CHM treating NAFLD and its complications by Heat-Clearing (Qing Re).

Active ingredients	Source (latin or English name/pinyin)	Efficacy of TCM theory	Experimental model	Molecule Mechanisms	Pathological mechanism	Ref.
14-Deoxy-11, 12-Didehydroandrographolide	Andrographis paniculata (Burm.f.) Nees [Acanthaceae]/Chuan-Xin-Lian	Clearing heat and detoxification	C57BL/6J mice fedHFHC model	Reduce cholesterol accumulation, antioxidant and anti-inflammatory activities	1,2	(69)
Isandrographolide			Wistar rats fed HFD model	Improve efficacy on hyperlipidemia and fat accumulation in the liver	2	(70)
Ursodeoxycholic acid	Bear bile/Xiong-Dan		SD rats fed HFD model	Inhibition of apoptosis and promotion of autophagy by activating AMPK pathway	4	(75)
			L02 cell stimulated with OA model	Regulate the AKT/mTOR/SREBP-1 signaling pathway	1,2	(76)
Genipin	Gardenia jasminoides J.Ellis [Rubiaceae]/Zhi-Zi		Aging SD rats model";"palmitate-treated L02 cell model	Inhibiting hepatic oxidative stress and mitochondrial dysfunction	1	(83)
			C57BL/6J mice fed HFD model	Reverses HFD-induced liver damage and inhibits UCP2-mediated pyroptosis	4	(79)
Geniposide			Tyloxapol- induced C57BL/6J mice model";"HepG2 cell induced by OA or PA model	Enhancing the ability of antioxidative stress and inflammation by up-regulating the protein expression of Nrf2/HO-1 and AMPK signaling pathways	1,3	(10)
			Free Fatty Acid-Treated HepG2 Cell model";"	Suppressed the intra- cellular lipid accumulation by increasing the expression of PPARα	2	(82)
			C57BL/6 mice fed HFD model	Down-regulate RHOA/ROCK signal and improve intestinal Barrier	8	(81)
			Wistar Rats fed HFD model	Improve fatty acid metabolism by regulating the AMPK–Malonyl-CoA-FFA axis.	2	(80)
GJ extract			LPS induced BV-2 cells model";"LPS induced AD rats model	Improve inflammation by suppression of JNK2/1 signaling pathways	2,3	(77)
			SD rats fed HFD model	Reduction of TNF-α and IL6 in adipose tissue	3	(78)
Cassia semen ethanol extract	Senna tora (L.) Roxb. [Fabaceae]/Jue-Ming-Zi		Wistar rats fed HFD model	The antioxidant effect through increased the levels of TNF-α, IL-6, IL-8 and MDA	1	(84)
Baicalin	Scutellaria baicalensis Georgi [Lamiaceae]/Huang-Qin	Heat-clearing and Fire-draining	KK-A mice and C57BL/6J mice OA-induced model";"Sodium oleate-induced cell model	Prevent lipotoxicity through the AMPK-mediated SREBP signaling pathway.	2,3	(86)
			HepG2 cells treated FFA model	Reduce proptosis of hepatocyte by blocking NLRP3–GSDMD signaling	4	(11)
Baicalein			Oleic acid-induced HepG2 cells";" HFD-induced mice model	Activating AMPK and suppressing SREBP1 cleavage	1,2	(89)
			MCD diet-induced mice C57BL/6J model	Attenuate lipid metabolism, inflammation and fibrosis in mice by suppressing key regulators such as SREBP-1c, FASN, PPARα ";"TNF-α, IL-1β ";"α-SMA and TGF-β1 and Col1A1	2,3,5	(90)
Oxymatrine	Sophora flavescens Aiton [Fabaceae]/Ku-Shen		High-fructose diet-induced Wistar rats’ model	Increase the mRNA and protein levels of PPA Rα 、CPT1and MTTP to decrease lipid accumulation in the liver	2	(93)
Matrine			C57BL/6J mice fed MCD model	Enhancing HSP72 and downregulating MTOR to improve inflammation and fibrosis	3,5	(96)
			C57BL/6J mice fed HFD model	Regulation of SERCA pathway reduces ER stress and mitochondrial dysfunction	1	(95)
Water Extract of Artemisia annua L.	Artemisia annua L. [Asteraceae]/Qing-Hao	Clearing deficiency heat	HepG2 cell treated with oleic acid or tert-butylhydroperoxide model; C57BL/6J mice fed HFD model	ImproveLipid Accumulation and Oxidative Stress	1,2	(101)
Rhein	Rheum palmatum L. [Polygonaceae]/Da-Huang	Purging fire and detoxification	C57BL/6J mice fed HFD model; LXR^-^/-Mice model; Hepa1–6 cell lines and Splenic mononuclear cell	Down-regulate lipogenesis through LXR-mediated SREBP-1c and shift the imbalanced Th1/Th2 response in the liver by modulation of cytokine signaling	2,7	(105)
			3T3-L1 adipocytes and HepG2 cells ";"C57BL/6J mice fed HFD model";"db/db Mice";"LXRα/β knockout (LXR–/–)mice	Activate the UCP1 gene by antagonizing the repressive effect of LXR on UCP1 expression to Improve lipid metabolism	2,6	(103)
Rhein lysinate			C57BL/J mice and KK/hlj mice fed HFD model	Decrease the expression of TNF-α, IL-6, NF-κB, SREBP-1c and Fas in liver	2,3	(106)
Emodin			Zebrafish fed Egg yolk powder model	Reduce hepatic lipogenesis by Regulation of AMPK Signaling Pathway	2	(111)
			HepG2 cell treated with FAA";"SD rats fed HFD model	Alleviate hepatic lipid accumulation by inhibiting SREBP1 activity *via* the camkk-AMPK-mTOR-p70s6k signaling pathway	2	(109)
			SD rats fed liquid fructose model	Improve the lipid accumulation through the ERS–SREBP 1c pathway	1,2	(110)
–			SD rats fed HFD model	Attenuate excess fat accumulation by promoting the activity of AMPK and decreasing the gene expression of the biosynthesis of fatty acids and TG.	2	(112)
–	Aloe vera (L.) Burm.f. [Asphodelaceae]/Lu-Hui		SD rats fed HFHFD model	Reduce oxidative stress, liver inflammation	2,3	(18)
Aloin			Nrf2 KO (Nrf2−/−) C57BL/6J mice model fed CDAAH diet model	Enhance antioxidant, anti-inflammatory and anti-apoptotic activity by activating Nrf2/HO-1 signaling	2,3,4	(116)

sThe current pathological mechanism of NAFLD:1.oxidative stress (OS) or ER stress, 2. abnormal lipid metabolism, 3. inflammation, 4.cell regeneration, 5.fibrosis, 6.genetic predisposition, 7.innate immune disorder, 8.intestinal flora imbalance 9. insulin resistance. CDAAH diet: choline-deficient, L-amino acid- defined, high-fat (CDAAH) diet; HFHFD:high-fat high-fructose diet

**Figure 5:**
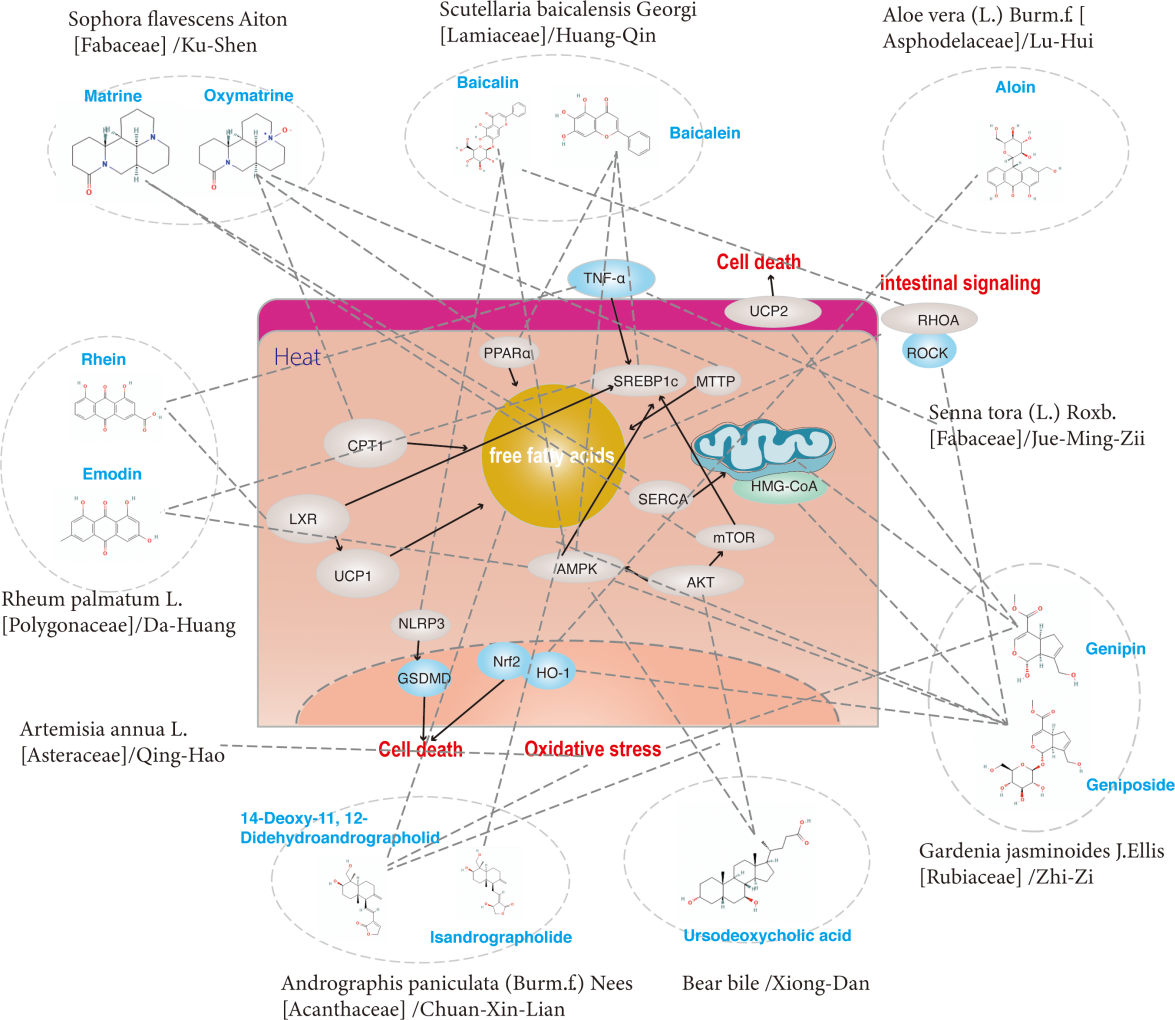
The regulatory effects on pathways induced by a group of ingredients from Heat-Clearing (Qing Re) herbs.

**Figure 6:**
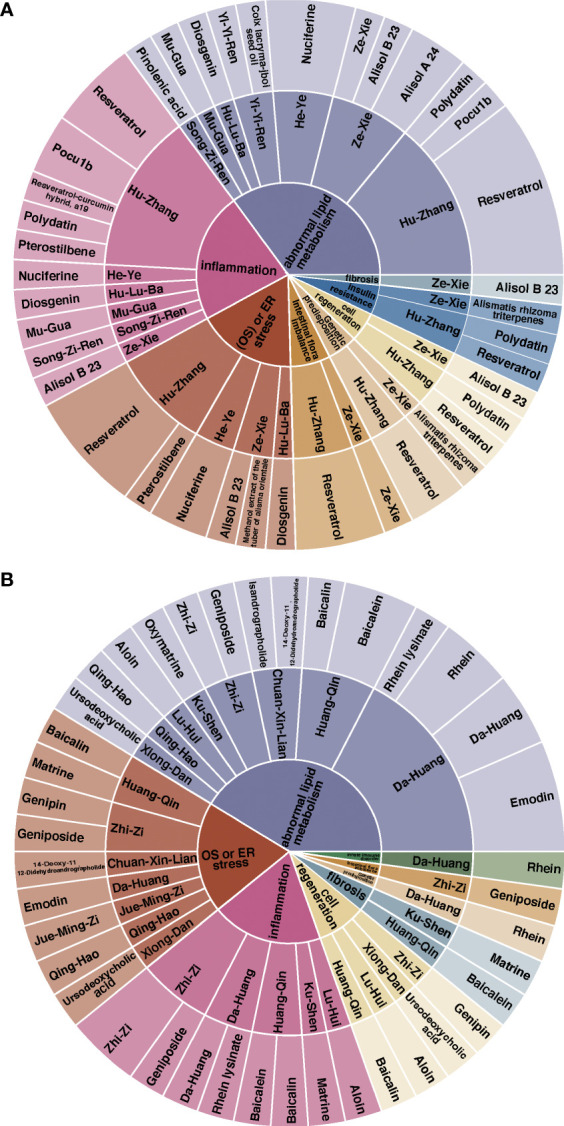
The relationship between **(A)** anti-dampness herbs and their active components, **(B)** clearing-heat herbs and their active components and the current pathological factors of NAFLD. The inner part is the pathological mechanism of NAFLD, the middle represents the corresponding herbs, and the outside is their active components. The colors represent the different molecular mechanisms of pathology and the size represents the proportion of the current study. The names of the individual herbs are given in Chinese and the corresponding English names are detailed in **Tables 2**, **3**.

Clearing heat is one of the important functions of traditional Chinese herbs, which can resist inflammation, reduce immune response and so on. Amongst all the traditional herbs that have been reported and elucidated the underlying mechanism for the treatment of non-alcoholic fatty liver disease, those drugs that can clear heat are Andrographis paniculata (Burm.f.) Nees [Acanthaceae]/Chuan-Xin-Lian, Bear bile/Xiong-Dan, Gardenia jasminoides J.Ellis [Rubiaceae]/Zhi-Zi, Senna tora (L.) Roxb. [Fabaceae]/Jue-Ming-Zi, Scutellaria baicalensis Georgi [Lamiaceae]/Huang-Qin, Sophora flavescens Aiton [Fabaceae]/Ku-Shen, Artemisia annua L. [Asteraceae]/Qing-Hao, Rheum palmatum L. [Polygonaceae]/Da-Huang and Aloe vera (L.) Burm.f. [Asphodelaceae]/Lu-Hui and its active monomer (**Table 3**). To be exact, 14-Deoxy-11, 12-Didehydroandrographolide, Ursodeoxycholic acid, Genipin, Geniposide, Emodin and Baicalin were proven reducers of oxidative and ER stress in NAFLD models. In addition, 14-Deoxy-11, 12-Didehydroandrographolide, Isandrographolide, Ursodeoxycholic acid, Geniposide, Baicalin, Baicalein, Oxymatrine, Rhein, Aloin and Emodin could improve NAFLD by reducing abnormal lipid accumulation in the liver (**Figures 5**, **6B**). Furthermore, Geniposide, Baicalin, Baicalein, Matrine, Rhein lysinate and Aloin were proven to alleviate liver inflammation in animal models. Moreover, Ursodeoxycholic acid, Baicalin, Aloin and Genipin could affect hepatocyte regeneration through different mechanisms.

Taking the above together, herbs and their active components that target one or 'multiple hits' of the `multiple hits' pathogenesis processes could affect NAFLD. **Figure 6** shows that improving lipid metabolism, reducing ER or oxidative stress and improving inflammation are the main pathological mechanisms of current anti-DHS herbs, accounting for more than 75% of current studies. However, the current challenge is how to improve traditional Chinese medicine and its active ingredients in the treatment of NAFLD. we propose the following suggestions:

1) To better explore the mechanisms of herbs (which are already used in clinics) and their active components, preclinical studies based on the reverse-pharmacology-guided approach are needed. In terms of experimental models, more models that can simulate human NAFLD need to be developed. It’s necessary to consider mainly herbs selection, dose, route of administration and dosing regimen for herbs delivery design.
2) Multiple types of omics analysis such as metagenomics, transcriptomics, metabolomics and proteomics can be used for high-throughput screening of herbal medicines for target/molecular mechanism exploration. Specifically, 16s ribosomal RNA analysis enables metagenomic analysis of the gut microbiota, allowing observation of the effects of herbal medicines on the gut flora. Moreover, RNA sequencing (RNA-Seq) using next-generation sequencing technology helps to analyze the cellular transcriptome.
3) Several biological databases have been widely used to identify drugs with common mechanisms of action, adapt existing drugs for use, discover the molecular mechanisms of unknown drugs, and search for potential drugs for diseases. Connectivity Map (CMAP) is a database that stores the mRNA expression levels of cells treated with different small molecule drugs, depending on the degree of up- or down-regulation compared to the control group. Combined with the development of group technology such as RNA-seq, we can predict the effective components in traditional Chinese medicine by using CMAP. Exploring Chinese medicine is an uncertain, winding and arduous path that requires continuous learning with the help of modern technology such as bioinformatics.
4) To dig deeper into the ancient Chinese medicine prescriptions, clarify their chemical composition by using modern science and technology, establish the connection between the theoretical evidence of Chinese medicine and modern mechanisms, and find new treatment methods for NAFLD.

## Author contributions

ZZ, JZ, KW, and LY are involved in writing and revising articles. TW, KW, and LW are responsible for literature search. XK, YG, and XS are responsible for the design of review ideas and revision of articles. All authors contributed to the article and approved the submitted version.

## Funding

This work was supported by the National Natural Science Foundation of China (82074336 to XS, 81873582 to XK, 81874436 to YG) and Program of Shanghai 2020 Science and Technology Innovation Action Plan (20S21901600 to XS).

## Acknowledgments

We thank Zhou Shasha and Zhu Anning for their help in grammar and figure processing. Thanks to, Huang Star U, for her moral support.

## Conflict of interest

The authors declare that the research was conducted in the absence of any commercial or financial relationships that could be construed as a potential conflict of interest.

## Publisher's note

All claims expressed in this article are solely those of the authors and do not necessarily represent those of their affiliated organizations, or those of the publisher, the editors and the reviewers. Any product that may be evaluated in this article, or claim that may be made by its manufacturer, is not guaranteed or endorsed by the publisher.

## References

[1] Chalasani N Younossi Z Lavine JE Diehl AM Brunt EM Cusi K. The diagnosis and management of non-alcoholic fatty liver disease: Practice guideline by the American gastroenterological association, American association for the study of liver diseases, and American college of gastroenterology. Gastroenterology (2012) 142(7):1592–609. doi: 10.1053/j.gastro.2012.04.001 22656328

[2] Brown GT Kleiner DE. Histopathology of nonalcoholic fatty liver disease and nonalcoholic steatohepatitis. Metabolism (2016) 65(8):1080–6. doi: 10.1016/j.metabol.2015.11.008 PMC4889547 26775559

[3] Younossi ZM Koenig AB Abdelatif D Fazel Y Henry L Wymer M. Global epidemiology of nonalcoholic fatty liver disease-meta-analytic assessment of prevalence, incidence, and outcomes. Hepatology (2016) 64(1):73–84. doi: 10.1002/hep.28431 26707365

[4] Sayiner M Koenig A Henry L Younossi ZM. Epidemiology of nonalcoholic fatty liver disease and nonalcoholic steatohepatitis in the united states and the rest of the world. Clin Liver Dis (2016) 20(2):205. doi: 10.1016/j.cld.2015.10.001 27063264

[5] Than NN Newsome PN. A concise review of non-alcoholic fatty liver disease. Atherosclerosis (2015) 239(1):192–202. doi: 10.1016/j.atherosclerosis.2015.01.001 25617860

[6] Green CD Jump DB Olson LK. Elevated insulin secretion from liver X receptor-activated pancreatic beta-cells involves increased *de novo* lipid synthesis and triacylglyceride turnover. Endocrinology (2009) 150(6):2637–45. doi: 10.1210/en.2008-1039 PMC2689804 19228891

[7] Banini BA Sanyal AJ. Current and future pharmacologic treatment of nonalcoholic steatohepatitis. Curr Opin Gastroen (2017) 33(3):134–41. doi: 10.1097/MOG.0000000000000356 PMC5491795 28346237

[8] Rinella ME. Nonalcoholic fatty liver disease a systematic review. Jama-J Am Med Assoc (2015) 313(22):2263–73. doi: 10.1001/jama.2015.5370 26057287

[9] Hosseini H Teimouri M Shabani M Koushki M Babaei Khorzoughi R Namvarjah F. Resveratrol alleviates non-alcoholic fatty liver disease through epigenetic modification of the Nrf2 signaling pathway. Int J Biochem Cell Biol (2020) 119:105667. doi: 10.1016/j.biocel.2019.105667 31838177

[10] Shen B Feng H Cheng J Li Z Jin M Zhao L. Geniposide alleviates non-alcohol fatty liver disease *via* regulating Nrf2/AMPK/mTOR signalling pathways. J Cell Mol Med (2020) 24(9):5097–108. doi: 10.1111/jcmm.15139 PMC7205797 32293113

[11] Shi H Zhang Y Xing J Liu L Qiao F Li J. Baicalin attenuates hepatic injury in non-alcoholic steatohepatitis cell model by suppressing inflammasome-dependent GSDMD-mediated cell pyroptosis. Int Immunopharmacol (2020) 81:106195. doi: 10.1016/j.intimp.2020.106195 32028242

[12] Zhang XY Kuang CB. Clinical observation on treatment of nonalcoholic fatty liver disease complicating hyperuricemia by zhifang I decoction. Zhong Xi Yi Jie He Xue Bao (2004) 2(4):265–267, 313. doi: 10.3736/jcim20040408 15339411

[13] Tang JM Liang HQ Wang HG Lin MT Zhang LM Chen SD. Efficacy of zaozhu yinchen recipe for treating non-alcoholic steatohepatitis and its effect on free fatty acid and TNF-alpha. Zhongguo Zhong Xi Yi Jie He Za Zhi (2016) 36(5):544–8. 27386644

[14] Li JX Wang YL Liu M Liu SN Xu CJ Zhao J. Treatment of nonalcoholic steatohepatitis by jianpi shugan recipe: a multi-center, randomized, controlled clinical trial. Zhongguo Zhong Xi Yi Jie He Za Zhi (2014) 34(1):15–9. 24520780

[15] Chen Z Wang P. Clinical distribution and molecular basis of traditional Chinese medicine ZHENG in cancer. Evidence-Based Complementary Altern Med (2012) 2012. doi: 10.1155/2012/783923 PMC3398674 22829858

[16] Organization WH. WHO international standard terminologies on traditional medicine in the western pacific region. (2007).

[17] Zhang GD Liu XX Liang JL Hu QM. The distribution pattern of traditional Chinese medicine syndromes in 549 patients with type 2 diabetes. Diabetes Metab Syndr Obes (2021) 14:2209–16. doi: 10.2147/DMSO.S295351 PMC8139680 34040406

[18] Klaikeaw N Wongphoom J Werawatganon D Chayanupatkul M Siriviriyakul P. Anti-inflammatory and anti-oxidant effects of aloe vera in rats with non-alcoholic steatohepatitis. World J Hepatol (2020) 12(7):363–77. doi: 10.4254/wjh.v12.i7.363 PMC7407916 32821335

[19] Chen M Ding Y Tong Z. Efficacy and safety of sophora flavescens (Kushen) based traditional Chinese medicine in the treatment of ulcerative colitis: Clinical evidence and potential mechanisms. Front Pharmacol (2020) 11. doi: 10.3389/fphar.2020.603476 PMC7758483 33362558

[20] Ludwig J Viggiano TR McGill DB Oh BJ. Nonalcoholic steatohepatitis: Mayo clinic experiences with a hitherto unnamed disease. Mayo Clin Proc (1980) 55(7):434–8. 7382552

[21] Dong H Lu F-e Zhao L. Chinese Herbal medicine in the treatment of nonalcoholic fatty liver disease. Chin J Integr Med (2012) 18(2):152–60. doi: 10.1007/s11655-012-0993-2 22311412

[22] Dai X Feng J Chen Y Huang S Shi X Liu X. Traditional Chinese medicine in nonalcoholic fatty liver disease: molecular insights and therapeutic perspectives. Chin Med (2021) 16(1):68. doi: 10.1186/s13020-021-00469-4 34344394 PMC8330116

[23] Wei HF Liu T Xing LJ Zheng PY Ji G. Distribution pattern of traditional Chinese medicine syndromes in 793 patients with fatty liver disease. Zhong Xi Yi Jie He Xue Bao (2009) 7(5):411–7. doi: 10.3736/jcim20090503 19435553

[24] GAO G LI S XUE J BAI Y HUI Y. Traditional Chinese medicine syndrome types and syndrome elements of nonalcoholic fatty liver disease. J Clin Hepatol (2021) 37(1):89–93. doi: 10.3969/j.issn.1001-5256.2021.01.018

[25] Unschuld PU Tessenow H. Huang Di nei jing su wen. University of California Press (2011).

[26] Wang ZJ Wang XH Li J Zheng SH Zhang FP Hao SL. The efficacy and safety of modified gegenqinlian fomular for advanced colorectal cancer (damp heat accumulation type): A multicenter randomized controlled trial. Med (Baltimore) (2021) 100(49):e27850. doi: 10.1097/MD.0000000000027850 PMC8663849 34889235

[27] Tian G Wu C Li J Liang B Zhang F Fan X. Network pharmacology based investigation into the effect and mechanism of modified sijunzi decoction against the subtypes of chronic atrophic gastritis. Pharmacol Res (2019) 144:158–66. doi: 10.1016/j.phrs.2019.04.012 30991106

[28] Ma L Zheng X Yang Y Wang J Xu Y Wang B. Epigenetic differences of chronic hepatitis b in different TCM syndromes: Protocol for a case-control, non-interventional, observational clinical study. Med (Baltimore) (2018) 97(39):e12452. doi: 10.1097/MD.0000000000012452 PMC6181568 30278525

[29] Chen Y Jiang TH Ru WZ Mao AW Liu Y. Objective tongue inspection on 142 liver cancer patients with damp-heat syndrome. Chin J Integr Med (2014) 20(8):585–90. doi: 10.1007/s11655-014-1756-z 24916806

[30] Chen FP Chang CM Wu TP Yang JL Kung YY Huang YH. Clinical efficacy of rong-Yang-Jyh-Gan-Tang on patients with chronic hepatitis c: A double-blinded randomized placebo-controlled crossover study. J Ethnopharmacol (2017) 196:1–8. doi: 10.1016/j.jep.2016.12.013 27965049

[31] Ding D Yan H Zhen X. Effects of Chinese herbs in children with henoch-schonlein purpura nephritis: a randomized controlled trial. J Tradit Chin Med (2014) 34(1):15–22. doi: 10.1016/S0254-6272(14)60048-0 25102685

[32] Zhang XX Chen WW She B Luo RJ Shi N Xue P. The efficacy and safety of jian-Wei-Qu-Tong pills for the treatment of chronic non-atrophic gastritis (spleen and stomach qi deficiency with damp-heat stasis syndrome): study protocol for a phase II, randomized controlled trial. Trials (2014) 15:272. doi: 10.1186/1745-6215-15-272 25002101 PMC4227091

[33] Liu D Yan J Yun M Yang M Luo Y Zhang J. Effect of sanhuangyilong decoction plus methotrexate on tumor necrosis factor alpha and interferon gamma in serum and synovial fluid in rheumatoid arthritis patients with symptom pattern of damp heat obstruction. J Tradit Chin Med (2016) 36(5):625–33. doi: 10.1016/S0254-6272(16)30082-6 29933531

[34] Zhu Y Ge XD Shi Y Guo JH Liu ZJ Zeng QQ. Efficacy and safety of numero sign I empirical prescription for chronic prostatitis in the treatment of type refractory chronic prostatitis. Zhonghua Nan Ke Xue (2018) 24(7):640–4. 30173449

[35] Dai YC Zheng L Zhang YL Chen X Chen DL Tang ZP. Effects of jianpi qingchang decoction on the quality of life of patients with ulcerative colitis: A randomized controlled trial. Med (Baltimore) (2017) 96(16):e6651. doi: 10.1097/MD.0000000000006651 PMC5406085 28422869

[36] Ting L Li-Ying WU Xiao-Mei Y Yao C Xue-Jiao DU Xue-Dan L. Curative effect of kangfuyan capsule combined with antibiotic treatment on pelvic inflammatory disease. Pak J Pharm Sci (2021) 34(6(Special):2479–85. 35039263

[37] J Z: Relationship between syndrome differentiation of nonalcoholic fatty liver, body mass index and blood fat. J Yunnan Univ Traditional Chin Med (2012) 35(6):28–30.

[38] XIE C Huang X Yang T. Analysis of the relationship between the common TCM syndrome types and the biochemical indexes of fatty liver. J Hunan Univ Chin Med (2018) 48(3):296–301. doi: 10.3969/j.issn.1674-070X.2018.03.016

[39] Zhang C Gu C. Study on metabolic disorder characteristics and TCM syndromes of non-alcoholic fatty liver. Shai Xi Zhong Yi (2014) 35(12):1597–9.

[40] Zhong X Du C Liu B Zhao J Wang W. Analysis of the relationship between insulin resistance, lipid metabolism and TCM syndromes in patients with non-alcoholic fatty liver. Beijing J Traditional Chin Med (2012) 31(7):493–5. doi: 10.16025/j.1674-1307.2012.07.003

[41] Zhang Y Tang K Deng Y Chen R Liang S Xie H. Effects of shenling baizhu powder herbal formula on intestinal microbiota in high-fat diet-induced NAFLD rats. BioMed Pharmacother (2018) 102:1025–36. doi: 10.1016/j.biopha.2018.03.158 29710519

[42] Xu J Wang R You S Zhang L Zheng P Ji G. Traditional Chinese medicine lingguizhugan decoction treating non-alcoholic fatty liver disease with spleen-yang deficiency pattern: Study protocol for a multicenter randomized controlled trial. Trials (2020) 21(1):512. doi: 10.1186/s13063-020-04362-7 32522273 PMC7288405

[43] Chen M Gu Y Huang F Zhong G Men L Liu Q. Effectiveness and safety of shugan jianpi (SGJP) formula in the treatment of nonalcoholic steatohepatitis (NASH): A protocol for systematic review and meta-analysis of randomized controlled trials. Med (Baltimore) (2021) 100(51):e28366. doi: 10.1097/MD.0000000000028366 PMC8702140 34941156

[44] Dang Y Xu J Yang Y Li C Zhang Q Zhou W. Ling-gui-zhu-gan decoction alleviates hepatic steatosis through SOCS2 modification by N6-methyladenosine. BioMed Pharmacother (2020) 127:109976. doi: 10.1016/j.biopha.2020.109976 32559839

[45] Dai L Xu J Liu B Dang Y Wang R Zhuang L. Lingguizhugan decoction, a Chinese herbal formula, improves insulin resistance in overweight/obese subjects with non-alcoholic fatty liver disease: a translational approach. Front Med (2022). doi: 10.1007/s11684-021-0880-3 35471471

[46] Zhang A Sun H Qiu S Wang X. Advancing drug discovery and development from active constituents of yinchenhao tang, a famous traditional chinese medicine formula. Evid Based Complement Alternat Med (2013) 2013:257909. doi: 10.1155/2013/257909 24191164 PMC3804150

[47] Lee TY Chang HH Lo WC Lin HC. Alleviation of hepatic oxidative stress by Chinese herbal medicine yin-Chen-Hao-Tang in obese mice with steatosis. Int J Mol Med (2010) 25(6):837–44. doi: 10.3892/ijmm_00000412 20428786

[48] Han R Qiu H Zhong J Zheng N Li B Hong Y. Si Miao formula attenuates non-alcoholic fatty liver disease by modulating hepatic lipid metabolism and gut microbiota. Phytomedicine (2021) 85:153544. doi: 10.1016/j.phymed.2021.153544 33773192

[49] Klayman DL. Qinghaosu (artemisinin): an antimalarial drug from China. Science (1985) 228(4703):1049–55. doi: 10.1126/science.3887571 3887571

[50] Soignet SL Maslak P Wang Z-G Jhanwar S Calleja E Dardashti LJ. Complete remission after treatment of acute promyelocytic leukemia with arsenic trioxide. New Engl J Med (1998) 339(19):1341–8. doi: 10.1056/NEJM199811053391901 9801394

[51] Zhang CH Sheng JQ Xie WH Luo XQ Xue YN Xu GL. Mechanism and basis of traditional Chinese medicine against obesity: Prevention and treatment strategies. Front Pharmacol (2021) 12:615895. doi: 10.3389/fphar.2021.615895 33762940 PMC7982543

[52] Xu X Li L Zhang Y Lu X Lin W Wu S. Hypolipidemic effect of alisma orientale (Sam.) juzep on gut microecology and liver transcriptome in diabetic rats. PloS One (2020) 15(10):e0240616. doi: 10.1371/journal.pone.0240616 33035272 PMC7546448

[53] Zhou X Ren Q Wang B Fang G Ling Y Li X. Alisol a 24-acetate isolated from the alismatis rhizoma improves hepatic lipid deposition in hyperlipidemic mice by ABCA1/ABCG1 pathway. J Nanosci Nanotechnol (2019) 19(9):5496–502. doi: 10.1166/jnn.2019.16592 30961702

[54] Xu F Yu H Lu C Chen J Gu W. The cholesterol-lowering effect of alisol acetates based on HMG-CoA reductase and its molecular mechanism. Evid Based Complement Alternat Med (2016) 2016:4753852. doi: 10.1155/2016/4753852 27872650 PMC5107224

[55] Lou HX Fu WC Chen JX Li TT Jiang YY Liu CH. Alisol a 24-acetate stimulates lipolysis in 3 T3-L1 adipocytes. BMC Complement Med Ther (2021) 21(1):128. doi: 10.1186/s12906-021-03296-0 33888116 PMC8063434

[56] Law BY Wang M Ma DL Al-Mousa F Michelangeli F Cheng SH. A novel inhibitor of the sarcoplasmic/endoplasmic reticulum Ca(2+) ATPase pump, induces autophagy, endoplasmic reticulum stress, and apoptosis. Mol Cancer Ther (2010) 9(3):718–30. doi: 10.1158/1535-7163.MCT-09-0700 20197400

[57] Meng Q Duan XP Wang CY Liu ZH Sun PY Huo XK. Alisol b 23-acetate protects against non-alcoholic steatohepatitis in mice *via* farnesoid X receptor activation. Acta Pharmacol Sin (2017) 38(1):69–79. doi: 10.1038/aps.2016.119 27773935 PMC5220543

[58] Jia XK Huang JF Huang XQ Li XY Huang MQ Zhu HC. Alismatis rhizoma triterpenes alleviate high-fat diet-induced insulin resistance in skeletal muscle of mice. Evid Based Complement Alternat Med (2021) 2021:8857687. doi: 10.1155/2021/8857687 33623531 PMC7875633

[59] Guo F Yang X Li X Feng R Guan C Wang Y. Nuciferine prevents hepatic steatosis and injury induced by a high-fat diet in hamsters. PloS One (2013) 8(5):e63770. doi: 10.1371/journal.pone.0063770 23691094 PMC3655021

[60] Zhang DD Zhang JG Wu X Liu Y Gu SY Zhu GH. Nuciferine downregulates per-Arnt-Sim kinase expression during its alleviation of lipogenesis and inflammation on oleic acid-induced hepatic steatosis in HepG2 cells. Front Pharmacol (2015) 6:238. doi: 10.3389/fphar.2015.00238 26539118 PMC4612658

[61] Cui H Li Y Cao M Liao J Liu X Miao J. Untargeted metabolomic analysis of the effects and mechanism of nuciferine treatment on rats with nonalcoholic fatty liver disease. Front Pharmacol (2020) 11:858. doi: 10.3389/fphar.2020.00858 32581811 PMC7295953

[62] Xue WL Li XS Zhang J Liu YH Wang ZL Zhang RJ. Effect of trigonella foenum-graecum (fenugreek) extract on blood glucose, blood lipid and hemorheological properties in streptozotocin-induced diabetic rats. Asia Pac J Clin Nutr (2007) 16 Suppl 1:422–6. 17392143

[63] Fang K Wu F Chen G Dong H Li J Zhao Y. Diosgenin ameliorates palmitic acid-induced lipid accumulation *via* AMPK/ACC/CPT-1A and SREBP-1c/FAS signaling pathways in LO2 cells. BMC Complement Altern Med (2019) 19(1):255. doi: 10.1186/s12906-019-2671-9 31519174 PMC6743105

[64] Chiang H Lu HF Chen JC Chen YH Sun HT Huang HC. Adlay seed (Coix lacryma-jobi l.) extracts exhibit a prophylactic effect on diet-induced metabolic dysfunction and nonalcoholic fatty liver disease in mice. Evid Based Complement Alternat Med (2020) 2020:9519625. doi: 10.1155/2020/9519625 32595752 PMC7275964

[65] Gu L Zhang Y Zhang S Zhao H Wang Y Kan D. Coix lacryma-jobi seed oil reduces fat accumulation in nonalcoholic fatty liver disease by inhibiting the activation of the p-AMPK/SePP1/apoER2 pathway. J Oleo Sci (2021) 70(5):685–96. doi: 10.5650/jos.ess20255 33840662

[66] Deenin W Malakul W Boonsong T Phoungpetchara I Tunsophon S. Papaya improves non-alcoholic fatty liver disease in obese rats by attenuating oxidative stress, inflammation and lipogenic gene expression. World J Hepatol (2021) 13(3):315–27. doi: 10.4254/wjh.v13.i3.315 PMC8006076 33815675

[67] Zhang J Zhang SD Wang P Guo N Wang W Yao LP. Pinolenic acid ameliorates oleic acid-induced lipogenesis and oxidative stress *via* AMPK/SIRT1 signaling pathway in HepG2 cells. Eur J Pharmacol (2019) 861:172618. doi: 10.1016/j.ejphar.2019.172618 31430456

[68] Lee AR Han SN. Pinolenic acid downregulates lipid anabolic pathway in HepG2 cells. Lipids (2016) 51(7):847–55. doi: 10.1007/s11745-016-4149-6 27084371

[69] Liu YT Chen HW Lii CK Jhuang JH Huang CS Li ML. A diterpenoid, 14-Deoxy-11, 12-didehydroandrographolide, in andrographis paniculata reduces steatohepatitis and liver injury in mice fed a high-fat and high-cholesterol diet. Nutrients (2020) 12(2):523. doi: 10.3390/nu12020523 PMC7071475 32085637

[70] Toppo E Darvin SS Esakkimuthu S Nayak MK Balakrishna K Sivasankaran K. Effect of two andrographolide derivatives on cellular and rodent models of non-alcoholic fatty liver disease. BioMed Pharmacother (2017) 95:402–11. doi: 10.1016/j.biopha.2017.08.071 28863380

[71] Duan MX Zhou H Wu QQ Liu C Xiao Y Deng W. Andrographolide protects against HG-induced inflammation, apoptosis, migration, and impairment of angiogenesis *via* PI3K/AKT-eNOS signalling in HUVECs. Mediators Inflammation (2019) 2019:6168340. doi: 10.1155/2019/6168340 PMC6800917 31686985

[72] Gao J Peng S Shan X Deng G Shen L Sun J. Inhibition of AIM2 inflammasome-mediated pyroptosis by andrographolide contributes to amelioration of radiation-induced lung inflammation and fibrosis. Cell Death Dis (2019) 10(12):957. doi: 10.1038/s41419-019-2195-8 31862870 PMC6925222

[73] Cabrera D Wree A Povero D Solis N Hernandez A Pizarro M. Andrographolide ameliorates inflammation and fibrogenesis and attenuates inflammasome activation in experimental non-alcoholic steatohepatitis. Sci Rep (2017) 7(1):3491. doi: 10.1038/s41598-017-03675-z 28615649 PMC5471224

[74] Paumgartner G Beuers U. Ursodeoxycholic acid in cholestatic liver disease: mechanisms of action and therapeutic use revisited. Hepatology (2002) 36(3):525–31. doi: 10.1053/jhep.2002.36088 12198643

[75] Wu P Zhao J Guo Y Yu Y Wu X Xiao H. Ursodeoxycholic acid alleviates nonalcoholic fatty liver disease by inhibiting apoptosis and improving autophagy *via* activating AMPK. Biochem Biophys Res Commun (2020) 529(3):834–8. doi: 10.1016/j.bbrc.2020.05.128 32595039

[76] Hu J Hong W Yao KN Zhu XH Chen ZY Ye L. Ursodeoxycholic acid ameliorates hepatic lipid metabolism in LO2 cells by regulating the AKT/mTOR/SREBP-1 signaling pathway. World J Gastroenterol (2019) 25(12):1492–501. doi: 10.3748/wjg.v25.i12.1492 PMC6441910 30948912

[77] Lin WH Kuo HH Ho LH Tseng ML Siao AC Hung CT. Gardenia jasminoides extracts and gallic acid inhibit lipopolysaccharide-induced inflammation by suppression of JNK2/1 signaling pathways in BV-2 cells. Iran J Basic Med Sci (2015) 18(6):555–62. PMC4509950 26221479

[78] Tan Y Lao W Xiao L Wang Z Xiao W Kamal MA. Managing the combination of nonalcoholic fatty liver disease and metabolic syndrome with chinese herbal extracts in high-fat-diet fed rats. Evid Based Complement Alternat Med (2013) 2013:306738. doi: 10.1155/2013/306738 23476686 PMC3588405

[79] Zhong H Liu M Ji Y Ma M Chen K Liang T. Genipin reverses HFD-induced liver damage and inhibits UCP2-mediated pyroptosis in mice. Cell Physiol Biochem (2018) 49(5):1885–97. doi: 10.1159/000493651 30235442

[80] Liang HQ Lin MT Zhao X Zhou HH Wang HG Li GH. Mechanism of geniposide in improving free fatty acid metabolism in rats with non-alcoholic fatty liver disease. Zhongguo Zhong Yao Za Zhi (2016) 41(3):470–5. doi: 10.4268/cjcmm20160319 28868866

[81] Peng JH Leng J Tian HJ Yang T Fang Y Feng Q. Geniposide and chlorogenic acid combination ameliorates non-alcoholic steatohepatitis involving the protection on the gut barrier function in mouse induced by high-fat diet. Front Pharmacol (2018) 9:1399. doi: 10.3389/fphar.2018.01399 30618733 PMC6298419

[82] Kojima K Shimada T Nagareda Y Watanabe M Ishizaki J Sai Y. Preventive effect of geniposide on metabolic disease status in spontaneously obese type 2 diabetic mice and free fatty acid-treated HepG2 cells. Biol Pharm Bull (2011) 34(10):1613–8. doi: 10.1248/bpb.34.1613 21963504

[83] Guan L Feng H Gong D Zhao X Cai L Wu Q. Genipin ameliorates age-related insulin resistance through inhibiting hepatic oxidative stress and mitochondrial dysfunction. Exp Gerontol (2013) 48(12):1387–94. doi: 10.1016/j.exger.2013.09.001 24041487

[84] Meng Y Liu Y Fang N Guo Y. Hepatoprotective effects of cassia semen ethanol extract on non-alcoholic fatty liver disease in experimental rat. Pharm Biol (2019) 57(1):98–104. doi: 10.1080/13880209.2019.1568509 30757944 PMC6374930

[85] Song KH Lee SH Kim BY Park AY Kim JY. Extracts of scutellaria baicalensis reduced body weight and blood triglyceride in db/db mice. Phytother Res (2013) 27(2):244–50. doi: 10.1002/ptr.4691 22532505

[86] Chen Q Liu M Yu H Li J Wang S Zhang Y. Scutellaria baicalensis regulates FFA metabolism to ameliorate NAFLD through the AMPK-mediated SREBP signaling pathway. J Nat Med (2018) 72(3):655–66. doi: 10.1007/s11418-018-1199-5 29542003

[87] Yu M Qi B Xiaoxiang W Xu J Liu X. Baicalein increases cisplatin sensitivity of A549 lung adenocarcinoma cells *via* PI3K/Akt/NF-kappaB pathway. BioMed Pharmacother (2017) 90:677–85. doi: 10.1016/j.biopha.2017.04.001 28415048

[88] Yan W Ma X Zhao X Zhang S. Baicalein induces apoptosis and autophagy of breast cancer cells *via* inhibiting PI3K/AKT pathway *in vivo* and vitro. Drug Des Devel Ther (2018) 12:3961–72. doi: 10.2147/DDDT.S181939 PMC6248272 30510404

[89] Sun W Liu P Wang T Wang X Zheng W Li J. Baicalein reduces hepatic fat accumulation by activating AMPK in oleic acid-induced HepG2 cells and high-fat diet-induced non-insulin-resistant mice. Food Funct (2020) 11(1):711–21. doi: 10.1039/C9FO02237F 31909773

[90] Zhang J Zhang H Deng X Zhang N Liu B Xin S. Baicalin attenuates non-alcoholic steatohepatitis by suppressing key regulators of lipid metabolism, inflammation and fibrosis in mice. Life Sci (2018) 192:46–54. doi: 10.1016/j.lfs.2017.11.027 29158052

[91] Shao J Liu Y Wang H Luo Y Chen L. An integrated fecal microbiome and metabolomics in T2DM rats reveal antidiabetes effects from host-microbial metabolic axis of EtOAc extract from sophora flavescens. Oxid Med Cell Longev (2020) 2020:1805418. doi: 10.1155/2020/1805418 32566075 PMC7273480

[92] Dong Y Xi H Yu Y Wang Q Jiang K Li L. Effects of oxymatrine on the serum levels of T helper cell 1 and 2 cytokines and the expression of the s gene in hepatitis b virus s gene transgenic mice: a study on the anti-hepatitis b virus mechanism of oxymatrine. J Gastroenterol Hepatol (2002) 17(12):1299–306. doi: 10.1046/j.1440-1746.2002.02885.x 12423275

[93] Shi L Shi L Zhang H Hu Z Wang C Zhang D. Oxymatrine ameliorates non-alcoholic fatty liver disease in rats through peroxisome proliferator-activated receptor-alpha activation. Mol Med Rep (2013) 8(2):439–45. doi: 10.3892/mmr.2013.1512 23754536

[94] Xu X Ling Q Gao F He ZL Xie HY Zheng SS. Hepatoprotective effects of marine and kuhuang in liver transplant recipients. Am J Chin Med (2009) 37(1):27–34. doi: 10.1142/S0192415X09006643 19222109

[95] Gao X Guo S Zhang S Liu A Shi L Zhang Y. Matrine attenuates endoplasmic reticulum stress and mitochondrion dysfunction in nonalcoholic fatty liver disease by regulating SERCA pathway. J Transl Med (2018) 16(1):319. doi: 10.1186/s12967-018-1685-2 30458883 PMC6245862

[96] Mahzari A Li S Zhou X Li D Fouda S Alhomrani M. Matrine protects against MCD-induced development of NASH *via* upregulating HSP72 and downregulating mTOR in a manner distinctive from metformin. Front Pharmacol (2019) 10:405. doi: 10.3389/fphar.2019.00405 31068812 PMC6491841

[97] Feng X Cao S Qiu F Zhang B. Traditional application and modern pharmacological research of artemisia annua l. Pharmacol Ther (2020) 216:107650. doi: 10.1016/j.pharmthera.2020.107650 32758647

[98] Efferth T. From ancient herb to modern drug: Artemisia annua and artemisinin for cancer therapy. Semin Cancer Biol (2017) 46:65–83. doi: 10.1016/j.semcancer.2017.02.009 28254675

[99] Lee AS Hur HJ Sung MJ. The effect of artemisinin on inflammation-associated lymphangiogenesis in experimental acute colitis. Int J Mol Sci (2020) 21(21):8068. doi: 10.3390/ijms21218068 PMC7662347 33138094

[100] Cao Q Du H Fu X Duan N Liu C Li X. Artemisinin attenuated atherosclerosis in high-fat diet-fed ApoE-/- mice by promoting macrophage autophagy through the AMPK/mTOR/ULK1 pathway. J Cardiovasc Pharmacol (2020) 75(4):321–32. doi: 10.1097/FJC.0000000000000794 31895870

[101] Choi EY Choi JO Park CY Kim SH Kim D. Water extract of artemisia annua l Exhibits hepatoprotective effects through improvement of lipid accumulation and oxidative stress-induced cytotoxicity. J Med Food (2020) 23(12):1312–22. doi: 10.1089/jmf.2020.4696 33202166

[102] Khiveh A Hashempur MH Shakiba M Lotfi MH Shakeri A Kazemeini S. Effects of rhubarb (Rheum ribes l.) syrup on dysenteric diarrhea in children: a randomized, double-blind, placebo-controlled trial. J Integr Med (2017) 15(5):365–72. doi: 10.1016/S2095-4964(17)60344-3 28844213

[103] Sheng X Zhu X Zhang Y Cui G Peng L Lu X. Rhein protects against obesity and related metabolic disorders through liver X receptor-mediated uncoupling protein 1 upregulation in brown adipose tissue. Int J Biol Sci (2012) 8(10):1375–84. doi: 10.7150/ijbs.4575 PMC3492795 23139635

[104] Wang H Yang D Li L Yang S Du G Lu Y. Anti-inflammatory effects and mechanisms of rhein, an anthraquinone compound, and its applications in treating arthritis: A review. Nat Prod Bioprospect (2020) 10(6):445–52. doi: 10.1007/s13659-020-00272-y PMC7648819 33128198

[105] Sheng X Wang M Lu M Xi B Sheng H Zang YQ. Rhein ameliorates fatty liver disease through negative energy balance, hepatic lipogenic regulation, and immunomodulation in diet-induced obese mice. Am J Physiol Endocrinol Metab (2011) 300(5):E886–893. doi: 10.1152/ajpendo.00332.2010 21364120

[106] Wei J Zhen YZ Cui J He FL Shen T Hu G. Rhein lysinate decreases inflammation and adipose infiltration in KK/HlJ diabetic mice with non-alcoholic fatty liver disease. Arch Pharm Res (2016) 39(7):960–9. doi: 10.1007/s12272-016-0770-4 27277164

[107] Zhu T Zhang W Feng SJ Yu HP. Emodin suppresses LPS-induced inflammation in RAW264.7 cells through a PPARgamma-dependent pathway. Int Immunopharmacol (2016) 34:16–24. doi: 10.1016/j.intimp.2016.02.014 26910236

[108] Xia S Ni Y Zhou Q Liu H Xiang H Sui H. Emodin attenuates severe acute pancreatitis *via* antioxidant and anti-inflammatory activity. Inflammation (2019) 42(6):2129–38. doi: 10.1007/s10753-019-01077-z 31605249

[109] Wang S Li X Guo H Yuan Z Wang T Zhang L. Emodin alleviates hepatic steatosis by inhibiting sterol regulatory element binding protein 1 activity by way of the calcium/calmodulin-dependent kinase kinase-AMP-activated protein kinase-mechanistic target of rapamycin-p70 ribosomal S6 kinase signaling pathway. Hepatol Res (2017) 47(7):683–701. doi: 10.1111/hepr.12788 27492505

[110] Li X Xu Z Wang S Guo H Dong S Wang T. Emodin ameliorates hepatic steatosis through endoplasmic reticulum-stress sterol regulatory element-binding protein 1c pathway in liquid fructose-feeding rats. Hepatol Res (2016) 46(3):E105–117. doi: 10.1111/hepr.12538 26031413

[111] Yu L Gong L Wang C Hu N Tang Y Zheng L. Radix polygoni multiflori and its main component emodin attenuate non-alcoholic fatty liver disease in zebrafish by regulation of AMPK signaling pathway. Drug Des Devel Ther (2020) 14:1493–506. doi: 10.2147/DDDT.S243893 PMC7167271 32346285

[112] Yang M Li X Zeng X Ou Z Xue M Gao D. Rheum palmatum l Attenuates high fat diet-induced hepatosteatosis by activating AMP-activated protein kinase. Am J Chin Med (2016) 44(3):551–64. doi: 10.1142/S0192415X16500300 27109162

[113] Gao Y Kuok KI Jin Y Wang R. Biomedical applications of aloe vera. Crit Rev Food Sci Nutr (2019) 59(sup1):S244–56. doi: 10.1080/10408398.2018.1496320 29999415

[114] Sun R Zhai R Ma C Miao W. Combination of aloin and metformin enhances the antitumor effect by inhibiting the growth and invasion and inducing apoptosis and autophagy in hepatocellular carcinoma through PI3K/AKT/mTOR pathway. Cancer Med (2020) 9(3):1141–51. doi: 10.1002/cam4.2723 PMC6997051 31830378

[115] Birari L Wagh S Patil KR Mahajan UB Unger B Belemkar S. Aloin alleviates doxorubicin-induced cardiotoxicity in rats by abrogating oxidative stress and pro-inflammatory cytokines. Cancer Chemother Pharmacol (2020) 86(3):419–26. doi: 10.1007/s00280-020-04125-w 32812061

[116] Xu Q Fan Y Loor JJ Liang Y Lv H Sun X. Aloin protects mice from diet-induced non-alcoholic steatohepatitis *via* activation of Nrf2/HO-1 signaling. Food Funct (2021) 12(2):696–705. doi: 10.1039/D0FO02684K 33410857

[117] Zeng XX Bian ZX Wu TX Fu SF Ziea E Woon WT. Traditional Chinese medicine syndrome distribution in chronic hepatitis b populations: a systematic review. Am J Chin Med (2011) 39(6):1061–74. doi: 10.1142/S0192415X11009408 22083981

[118] Jang MK Han YR Nam JS Han CW Kim BJ Jeong HS. Protective effects of alisma orientale extract against hepatic steatosis *via* inhibition of endoplasmic reticulum stress. Int J Mol Sci (2015) 16(11):26151–65. doi: 10.3390/ijms161125944 PMC4661803 26540043

[119] Izdebska M Piatkowska-Chmiel I Korolczuk A Herbet M Gawronska-Grzywacz M Gieroba R. The beneficial effects of resveratrol on steatosis and mitochondrial oxidative stress in HepG2 cells. Can J Physiol Pharmacol (2017) 95(12):1442–53. doi: 10.1139/cjpp-2016-0561 28759727

[120] Chen XX Xu YY Wu R Chen Z Fang K Han YX. Resveratrol reduces glucolipid metabolic dysfunction and learning and memory impairment in a NAFLD rat model: Involvement in regulating the imbalance of nesfatin-1 abundance and copine 6 expression. Front Endocrinol (Lausanne) (2019) 10:434. doi: 10.3389/fendo.2019.00434 31338065 PMC6629830

[121] Huang Y Lang H Chen K Zhang Y Gao Y Ran L. Resveratrol protects against nonalcoholic fatty liver disease by improving lipid metabolism and redox homeostasis *via* the PPARalpha pathway. Appl Physiol Nutr Metab (2020) 45(3):227–39. doi: 10.1139/apnm-2019-0057 31173696

[122] Zhang Y Chen ML Zhou Y Yi L Gao YX Ran L. Resveratrol improves hepatic steatosis by inducing autophagy through the cAMP signaling pathway. Mol Nutr Food Res (2015) 59(8):1443–57. doi: 10.1002/mnfr.201500016 25943029

[123] Izdebska M Herbet M Gawronska-Grzywacz M Piatkowska-Chmiel I Korga A Sysa M. Resveratrol limits lipogenesis and enhance mitochondrial activity in HepG2 cells. J Pharm Pharm Sci (2018) 21(1):504–15. doi: 10.18433/jpps29994 30522586

[124] Chen M Hou P Zhou M Ren Q Wang X Huang L. Resveratrol attenuates high-fat diet-induced non-alcoholic steatohepatitis by maintaining gut barrier integrity and inhibiting gut inflammation through regulation of the endocannabinoid system. Clin Nutr (2020) 39(4):1264–75. doi: 10.1016/j.clnu.2019.05.020 31189495

[125] Wang P Wang J Li D Ke W Chen F Hu X. Targeting the gut microbiota with resveratrol: a demonstration of novel evidence for the management of hepatic steatosis. J Nutr Biochem (2020) 81:108363. doi: 10.1016/j.jnutbio.2020.108363 32388250

[126] Wang P Li D Ke W Liang D Hu X Chen F. Resveratrol-induced gut microbiota reduces obesity in high-fat diet-fed mice. Int J Obes (Lond) (2020) 44(1):213–25. doi: 10.1038/s41366-019-0332-1 30718820

[127] Hajighasem A Farzanegi P Mazaheri Z. Effects of combined therapy with resveratrol, continuous and interval exercises on apoptosis, oxidative stress, and inflammatory biomarkers in the liver of old rats with non-alcoholic fatty liver disease. Arch Physiol Biochem (2019) 125(2):142–9. doi: 10.1080/13813455.2018.1441872 29463133

[128] Ji G Wang Y Deng Y Li X Jiang Z. Resveratrol ameliorates hepatic steatosis and inflammation in methionine/choline-deficient diet-induced steatohepatitis through regulating autophagy. Lipids Health Dis (2015) 14:134. doi: 10.1186/s12944-015-0139-6 26498332 PMC4619480

[129] Badi RM Mostafa DG Khaleel EF Satti HH. Resveratrol protects against hepatic insulin resistance in a rat's model of non-alcoholic fatty liver disease by down-regulation of GPAT-1 and DGAT2 expression and inhibition of PKC membranous translocation. Clin Exp Pharmacol Physiol (2019) 46(6):545–55. doi: 10.1111/1440-1681.13074 30773673

[130] Hajighasem A Farzanegi P Mazaheri Z Naghizadeh M Salehi G. Effects of resveratrol, exercises and their combination on farnesoid X receptor, liver X receptor and sirtuin 1 gene expression and apoptosis in the liver of elderly rats with nonalcoholic fatty liver. PeerJ (2018) 6:e5522. doi: 10.7717/peerj.5522 30221089 PMC6136396

[131] Li L Hai J Li Z Zhang Y Peng H Li K. Resveratrol modulates autophagy and NF-kappaB activity in a murine model for treating non-alcoholic fatty liver disease. Food Chem Toxicol (2014) 63:166–73. doi: 10.1016/j.fct.2013.08.036 23978414

[132] Gomez-Zorita S Gonzalez-Arceo M Trepiana J Aguirre L Crujeiras AB Irles E. Comparative effects of pterostilbene and its parent compound resveratrol on oxidative stress and inflammation in steatohepatitis induced by high-fat high-fructose feeding. Antioxidants (Basel) (2020) 9(11):1042. doi: 10.3390/antiox9111042 PMC7690896 33114299

[133] Wu B Xiao Z Zhang W Chen H Liu H Pan J. A novel resveratrol-curcumin hybrid, a19, attenuates high fat diet-induced nonalcoholic fatty liver disease. BioMed Pharmacother (2019) 110:951–60. doi: 10.1016/j.biopha.2018.11.088 30625517

[134] Kim J Kim CS Jo K Lee IS Kim JH Kim JS. POCU1b, the n-butanol soluble fraction of polygoni cuspidati rhizoma et radix, attenuates obesity, non-alcoholic fatty liver, and insulin resistance Via inhibitions of pancreatic lipase, cAMP-dependent PDE activity, AMPK activation, and SOCS-3 suppression. Nutrients (2020) 12(12):3612. doi: 10.3390/nu12123612 PMC7759958 33255404

[135] Zhang J Tan Y Yao F Zhang Q. Polydatin alleviates non-alcoholic fatty liver disease in rats by inhibiting the expression of TNF-alpha and SREBP-1c. Mol Med Rep (2012) 6(4):815–20. doi: 10.3892/mmr.2012.1015 22858825

[136] Zhang Q Tan Y Zhang N Yao F. Polydatin supplementation ameliorates diet-induced development of insulin resistance and hepatic steatosis in rats. Mol Med Rep (2015) 11(1):603–10. doi: 10.3892/mmr.2014.2708 25333896

[137] Chen X Chan H Zhang L Liu X Ho IHT Zhang X. The phytochemical polydatin ameliorates non-alcoholic steatohepatitis by restoring lysosomal function and autophagic flux. J Cell Mol Med (2019) 23(6):4290–300. doi: 10.1111/jcmm.14320 PMC6533566 30973211

